# Additional New Cytotoxic Triquinane-Type Sesquiterpenoids Chondrosterins K–M from the Marine Fungus *Chondrostereum* sp

**DOI:** 10.3390/md14090157

**Published:** 2016-08-26

**Authors:** Lei Huang, Wen-Jian Lan, Rong Deng, Gong-Kan Feng, Qing-Yan Xu, Zhi-Yu Hu, Xiao-Feng Zhu, Hou-Jin Li

**Affiliations:** 1 School of Chemistry and Chemical Engineering, Sun Yat-sen University, Guangzhou 510275, China; marinehuangl@gmail.com; 2 School of Pharmaceutical Sciences, Sun Yat-sen University, Guangzhou 510006, China; lanwj@mail.sysu.edu.cn; 3 Guangdong Technology Research Center for Advanced Chinese Medicine, Guangzhou 510006, China; 4 State Key Laboratory of Oncology in South China, Collaborative Innovation Center for Cancer Medicine, Cancer Center, Sun Yat-sen University, Guangzhou 510060, China; dengrong@sysucc.org.cn (R.D.); fenggk@sysucc.org.cn (G.-K.F.); 5 School of Life Sciences, Xiamen University, Xiamen 361102, China; xuqingyan@xmu.edu.cn (Q.-Y.X.); huzhiyu@xmu.edu.cn (Z.-Y.H.)

**Keywords:** *Chondrostereum* sp., chondrosterin, hirsutane, sesquiterpenoid, cytotoxicity

## Abstract

By the method of ^1^H NMR prescreening and tracing the diagnostic proton signals of the methyl groups, three additional new triquinane-type sesquiterpenoids—chondrosterins K–M (**1**–**3**) and the known sesquiterpenoid anhydroarthrosporone (**4**)—were isolated from the marine fungus *Chondrostereum* sp. Their structures were elucidated on the basis of MS, 1D, and 2D NMR data. Chondrosterin K is a rare hirsutane sesquiterpenoid, in which a methyl group was migrated from C-2 to C-6 and has a double bond between C-2 and C-3. Compounds **1**–**3** showed significant cytotoxicities against various cancer cell lines in vitro.

## 1. Introduction

**Figure 1:**
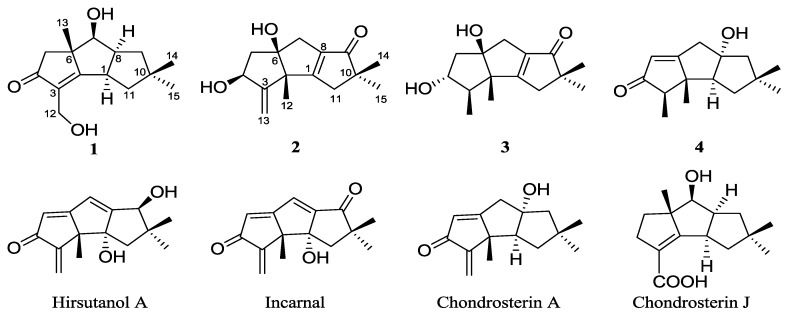
Chemical structures of compounds **1**–**4**, hirsutanol A, incarnal, and chondrosterins A and J.

In recent decades, a large number of novel compounds were isolated from the soft corals collected from the South China Sea, which have significant biological activities, such as antitumor, antivirus, anti-hypertension, anti-inflammatory, and analgesic [1]. However, the limited supply of the soft corals and their pharmaceutical lead compounds makes the drug development a very slow process; so, searching for alternative drug resources has become a crucial task. Marine fungi associated with the soft corals can be expected to metabolize biologically interesting and chemically diverse compounds and draw much attention [2,3]. Naturally occurring sesquiterpenoids with hirsutane frameworks are the typical metabolites of some fungi. Up to now, about fifty hirsutane-type compounds have been reported, and some of them have significant biological activities, such as antibacterial [4,5,6,7], cytotoxic [6,7,8,9], and antimalarial activities [9]. The fungal strain *Chondrostereum* sp. was isolated from the soft coral of *Sarcophyton tortuosum*. Previous isolation of metabolites led to the discovery of hirsutane sesquiterpenoid compounds, chondrosterins A–F [10,11], I–J [12], hirsutanols A [13], C [10], E [13], and F [13], incarnal [11], and arthrosporone [11]. Among them, hirsutanol A, incarnal, and chondrosterin A and J (Figure 1) showed potent cytotoxicities. Hirsutanol A inhibited the growth of cancer cells by increasing the level of reactive oxygen species (ROS) [14,15,16].

In our continued research project, the fungal strain *Chondrostereum* sp. was cultured in a large-scale glucose–peptone–yeast (GPY) medium. By tracing the characteristic proton NMR signals of the methyl groups around 1.00–1.40 ppm, chemical investigation of the extract led to the discovery of three new triquinane-type sesquiterpenoids, chondrosterins K–M (**1**–**3**), and the known sesquiterpenoid anhydroarthrosporone (**4**) (Figure 1) from the fungal culture extract. The structures of these metabolites were assigned on the basis of the detailed NMR and MS spectroscopic analysis. The isolation, structure identification, and cytotoxicities of these compounds are reported herein.

## 2. Results and Discussion

### 2.1. Structure Elucidation

**Figure 2:**
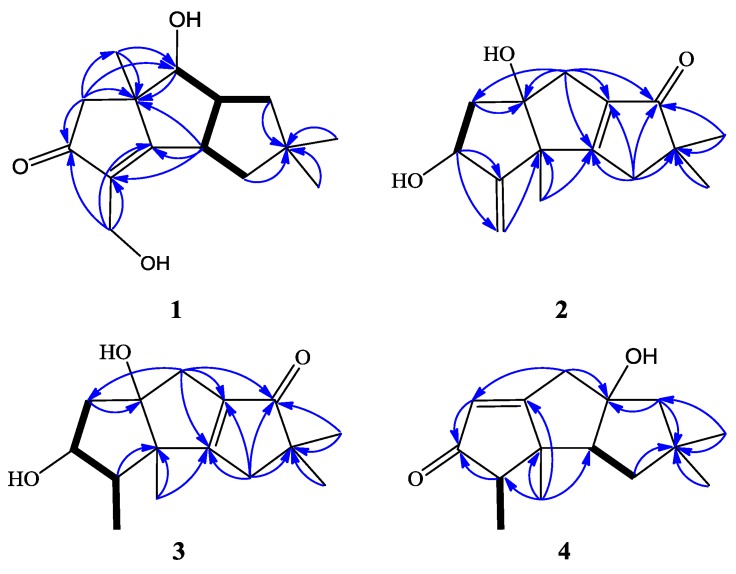
^1^H–^1^H COSY (bold line) and main HMBC (arrow) correlations of **1**–**4**.

**Figure 3:**
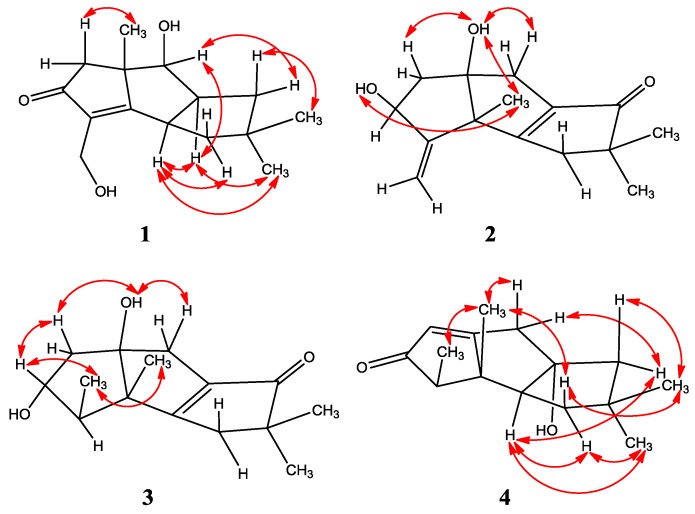
Selected key NOESY correlations of **1**–**4**.

Chondrosterin K (**1**) was isolated as a colorless oil. The HR-EI-MS data at *m/z* 250.1568 [M]^+^ (Supplementary Figure S1), along with the NMR data (Table 1 and Table 2, Supplementary Figures S2–S7) revealed the molecular formula of compound **1** to be C_15_H_22_O_3_, and the degrees of unsaturation are five. The UV absorption at λ_max_ 241 nm indicated a conjugated system formed by the carbonyl group and the double bond. So, this molecule must be tricyclic to count the five degrees of unsaturation. According to the ^1^H and ^13^C NMR and DEPT data (Table 1 and Table 2), compound **1** had three methyls, four methylenes, three methines, and five quaternary carbons. The typical functional groups included one carbonyl carbon (δ_C_ 210.1), one tetrasubstituted double bond (δ_C_ 186.4 and 135.2), three methyl group singlets (δ_H_ 1.01, 1.16, and 1.33), and two hydroxyl groups (δ_H_ 2.15, brs, 2H). The ^1^H–^1^H COSY cross-peaks of H-7 (CH, δ_H_ 4.05)/H-8 (CH, δ_H_ 3.16); H-8/H-9 (CH_2_, δ_H_ 1.92, 1.55), H-8/H-1 (CH, δ_H_ 3.47), and H-1/H-11 (CH_2_, δ_H_ 1.87, 1.63) deduced the fragment of –CHCH(CH_2_)CHCH_2_–. The HMBC correlations of H-1/C-2, H-1/C-6, H-5/C-4, H-5/C-6, H-5/C-13, H-7/C-6, H-7/C-13, H-9/C-10, H-11/C-10, H-12/C-3, H-12/C-4, H-13/C-5, H-13/C-6, H-13/C-7, H-14/C-10, and H-15/C-10 established the planar structure of compound **1** (Figure 2). The hydroxyl group at δ_H_ 2.15 (brs) connected with the methylene (CH_2_, δ_H_ 4.34, d, *J* = 13.2 Hz; 4.29, d, *J* = 13.2 Hz), and another hydroxyl group at δ_H_ 2.15 (brs) was connected to the methine group (C-7, δ_C_ 77.8). The NOESY correlations of H-1/H-8, H-1/H-11α, H-1/H-15, H-7/H-8, H-8/H-9α, and H-8/H-15 (Figure 3) revealed that H-1, H-7, H-8, and H-15 have an α-orientation. No NOESY correlation between H-13 and H-1, H-8 was observed, so C-13 was placed at the β position. Compound **1** is an unprecedented hirsutane-type sesquiterpenoid having a C-2/C-3 double bond in the molecule.

Chondrosterin L (**2**) was isolated as a yellowish oil. The molecular formula of compound **2** was determined as C_15_H_20_O_3_ based on the HR-EI-MS data at *m/z* 248.1410 [M]^+^ (Supplementary Figure S8) and the NMR data (Table 1 and Table 2, Supplementary Figures S9–S16). The degrees of unsaturation are six. The UV spectrum peak at λ_max_ 242 nm revealed an α,β-unsaturated carbonyl chromophore. Compound **2** contained three methyls, four methylenes, one methine, and seven quaternary carbons. The NMR data recorded in CDCl_3_ and Acetone-d_6_ are almost identical. The ^1^H–^1^H COSY spectrum recorded in CDCl_3_ showed the cross peak of H-4 (δ_H_ 4.45)/H-5 (δ_H_ 1.87 and 2.08) and established the structural fragment –CHCH_2_–. The carbonyl carbon at δ_C_ 209.7 connected the tetrasubstituted double bond (δ_C_ 184.8 and 139.1) forming a conjugated system and interpreted the UV absorption and the chemical shifts of the double bond at the lower shielding surrounding. The C-3 and C-13 formed one terminal double bond (δ_C_ 156.8, C; and δ_C_ 110.9, CH_2_). The HMBC correlations of H-4/C-3, H-4/C-13, H-5/C-6, H-7/C-6, H-7/C-8, H-7/C-9, H-11/C-1, H-11/C-9, H-11/C-10, H-12/C-1, H-12/C-2, H-13/C-2, H-14/C-9, H-14/C-10, H-15/C-9, and H-15/C-10 established the planar structure of **2** (Figure 2). C-4 and C-6 connected with the hydroxyl groups. Based on the NOESY correlations of 4-OH/H-12 (δ_H_ 1.31), 6-OH/H-5β (δ_H_ 1.87), 6-OH/H-7β (δ_H_ 2.43), and 6-OH/H-12 (Figure 3), 4-OH, 6-OH, and H-12 (CH_3_) were assigned as β positions.

Chondrosterin M (**3**) was isolated as a bright yellow oil. Its molecular formula was determined as C_15_H_22_O_3_ based on the HR-EI-MS data (*m/z* 250.1561 [M]^+^, Supplementary Figure S17), along with the NMR data (Table 1 and Table 3, Supplementary Figures S18–S25). Compound **3** has four methyls, three methylenes, two methines, and six quaternary carbons. ^1^H NMR data recorded in CDCl_3_ revealed three methyl groups with singlets (δ_H_ 1.12, 1.15, and 1.21), and one methyl group with doublet (δ_H_ 1.06) which connected with the methine carbon at C-3 (δ_C_ 52.1, δ_H_ 1.93); these are the diagnostic resonance signals of hirsutane sesquiterpenoids. By comparison, looking at the NMR data with compound **2**, quick identification was made that a fragment of the CH_3_CH– in compound **3** was substituted the terminal C=C double bond in compound **2**. The ^1^H–^1^H COSY cross-peaks of H-3 (CH, δ_H_ 1.93)/H-13(CH_3_, δ_H_ 1.06), H-3/H-4 (δ_H_ 3.63), and H-4/H-5 (CH, δ_H_ 1.91 and 2.28) (Figure 2) established the fragment CH_3_CHCHCH_2_–. The HMBC correlations of H-3/C-2, H-5/C-6, H-7/C-1, H-7/C-5, H-7/C-6, H-7/C-8, H-7/C-9, H-11/C-1, H-11/C-8, H-11/C-9, H-11/C-10, H-12/C-1, H-12/C-2, H-14/C-9, H-14/C-10, H-15/C-9, and H-15/C-10 (Figure 2) established the planar structure of **3**. The NOESY correlations of 6-OH (δ_H_ 2.53, brs)/H-5 (δ_H_ 1.91), 6-OH/H-7 (δ_H_ 2.45), 6-OH/H-12 (δ_H_ 1.21, CH_3_), H-4/H-13, and H-12/H-13 (Figure 3) deduced that 6-OH, H-12, and H-13 were placed at β position, whereas 4-OH was placed at α position.

Compound **4** has a molecular formula of C_15_H_22_O_2_ established by HR-EI-MS (*m/z* 234.1613) (Supplementary Figure S26) and NMR (Table 1 and Table 3, Supplementary Figures S27 and S28) data. It contains four methyls, three methylenes, three methines, and five quaternary carbons. The typical functional groups included one carbonyl carbon (δ_C_ 211.6), one tetrasubstituted double bond (δ_C_ 190.8 and 122.9), three methyl group singlets (δ_H_ 0.91, 1.11, and 1.20), and a methyl doublet at δ_H_ 1.08. The ^1^H–^1^H COSY spectra displayed the following cross-peaks: H-3 (δ_H_ 2.32)/H-13 (CH_3_, δ_H_ 1.08) and H-1(δ_H_ 2.38)/H-11(CH_2_, δ_H_ β: 1.46; α: 1.70), so the fragments CH_3_CH– and –CHCH_2_– were established. The HMBC correlations of H-3/C-4, H-5/C-4, H-7/C-5, H-7/C-6, H-7/C-8, H-9/C-8, H-9/C-10, H-11/C-10, H-12/C-1, H-12/C-2, H-12/C-3, H-12/C-6, H-14/C-9, H-14/C-10, H-14/C-11, H-15/C-9, H-15/C-10, and H-15/C-11 (Figure 2) established the planar structure of **6**. The NOESY correlations of H-12/H-7β (δ_H_ 2.71), H-12/H-11β (δ_H_ 1.46), H-12/H-13, and H-14/H-11β (Figure 2) established C-12, C-13, and C-14 as β-oriented. In addition, NOESY correlations between H-1/H-9α (δ_H_ 1.86), H-1/H-11α (δ_H_ 1.70), and H-1/H-15 (Figure 3) allowed assignment of H-1 and H-15 in α-orientation. Compound **4** was identified as anhydroarthrosporone, which was firstly isolated by Amouzou E and co-workers from a basidiomycete fungus *Ceratocystis ulmi* [17]. Our NMR data are obviously different from the reference data, although both of them were recorded in the same solvent (CDCl_3_). For example, our ^13^C NMR data of C-1, C-3, C-6, C-7, and C-9 are 63.4, 57.7, 190.8, 43.9, and 55.9, respectively. As a comparison, the corresponding reference values are 57.7, 63.4, 177.0, 55.9, and 44.0, respectively [17].

### 2.2. Biological Evaluation

Seven cancer cell lines were used to examine the cytotoxicities of compounds **1**–**4** in vitro. This assay revealed that **1**–**3** had significant cytotoxic effects (Table 4). In contrast, **4** were apparently inactive in this assay (IC_50_ values > 100 μM). Hirsutanol A was used as a positive control.

## 3. Materials and Methods

### 3.1. General Experimental Procedures

Preparative HPLC was performed using a Shimadzu LC-20AT HPLC pump (Shimadzu Corporation, Nakagyo-ku, Kyoto, Japan) equipped with an SPD-20A dual λ absorbance detector (Shimadzu Corporation, Nakagyo-ku, Kyoto, Japan) and a Shim-pack PRC-ODS HPLC column (250 mm × 20 mm, Shimadzu Corporation, Nakagyo-ku, Kyoto, Japan). Optical rotations were measured using a Schmidt and Haensch Polartronic HNQW5 optical rotation spectrometer (SCHMIDT + HAENSCH GmbH & Co., Berlin, Germany). UV spectra were recorded on a Shimadzu UV-VIS-NIR spectrophotometer (Shimadzu Corporation, Nakagyo-ku, Kyoto, Japan). IR spectra were recorded on a PerkinElmer Frontier FT-IR spectrophotometer (PerkinElmer Inc., Waltham, MA, USA). 1D and 2D NMR spectra were recorded on Bruker Avance III 400 and IIIT 600 HD spectrometers (Bruker BioSpin AG, Industriestrasse 26, Fällanden, Switzerland). The chemical shifts are relative to the residual solvent signals (CDCl_3_: δ_H_ 7.26 and δ_C_ 77.0; acetone-*d*_6_: δ_H_ 2.05 and δ_C_ 29.92). The low- and high-resolution EI mass spectra were obtained on Thermo DSQ and Thermo MAT95XP mass spectrometers (Thermo Fisher Scientific, Waltham, MA, USA), respectively.

### 3.2. Fungal Material

The marine fungus *Chondrostereum* sp. was isolated from the inner tissue of a soft coral of the species *Sarcophyton tortuosum* collected from the Hainan Sanya National Coral Reef Reserve, China. This fungal strain was deposited at School of Chemistry and Chemical Engineering, Sun Yat-sen University, Guangzhou 510275, China, and maintained in sterile aqueous solution of 15% (*v/v*) glycerol at −80 °C.

### 3.3. Fermentation, Extraction, and Isolation

The mycelia of *Chondrostereum* sp. were aseptically transferred to 500 mL Erlenmeyer flasks containing 200 mL of the sterilized GPY (glucose 10 g/L, peptone 5 g/L, yeast extract 2 g/L, NaCl 23 g/L) liquid medium. The flasks were then incubated at 28 °C on a rotary shaker (120 rpm) for 20 days. The cultures (200 L) were filtered through clean cheese cloth. The filtrate was extracted with ethyl acetate four times. The extract (31.6 g) was purified on a silica gel column with petroleum ether–EtOAc (100:0–0:100) and then EtOAc–MeOH (100:0–0:100) as the mobile phase to afford 12 fractions (code Fr. 1–Fr. 12). Fr. 6–7 were further purified by RP HPLC with an eluent of H_2_O–MeOH (40:60, *v/v*) to afford compounds **1** (8 mg), **2** (6 mg), and **3** (11 mg); compound **4** was obtained from Fr. 3 by Sephadex LH-20 gel column chromatography and repeated RP-HPLC eluted with H_2_O–MeCN (60:40, *v/v*).

Chondrosterin K (**1**): Colorless oil; [α]${}_{\text{D}}^{20}$ −31.1 (*c* 0.1, MeOH); UV (MeOH) λ_max_ (log ε) 241 nm (4.02); IR (KBr) ν_max_ 3356, 2926, 2856, 1690, 1650, 1513, 1455, 1367, 1262, 1228, 1109, 1058, 1033, 896, 829 cm^−1^; ^1^H and ^13^C NMR data, see Table 1; LR-EI-MS *m/z* 250, 232, 217, 199, 175, 123, 91, 77, 55; HR-EI-MS *m/z* 250.1568 [M]^+^ (calcd. for C_15_H_22_O_3_, 250.1563), 232.1456 [M − H_2_O]^+^ (calcd. for C_15_H_20_O_2_, 232.1458).

Chondrosterin L (**2**): Yellowish oil; [α]${}_{\text{D}}^{20}$ +66.5 (*c* 0.1, MeOH); UV (MeOH) λ_max_ (log ε) 242 nm (3.86); IR (KBr) ν_max_ 3375, 2926, 2855, 1683, 1629, 1514, 1456, 1383, 1264, 1229, 1107, 1061, 996, 909, 831, 829 cm^−1^; ^1^H and ^13^C NMR data, see Table 1; LR-EI-MS *m/z* 250, 232, 217, 199, 175, 123, 91, 77, 55; HR-EI-MS *m/z* 248.1410 [M]^+^ (calcd. for C_15_H_20_O_3_, 248.1414).

Chondrosterin M (**3**): Yellowish oil; [α]${}_{\text{D}}^{20}$ −1.17 (*c* 0.1, MeOH); UV (MeOH) λ_max_ (log ε) 241 nm (3.46); IR (KBr) ν_max_ 3357, 2928, 2856, 1681, 1626, 1452, 1432, 1388, 1283, 1234, 1110, 1049, 998, 922 cm^−1^; ^1^H and ^13^C NMR data, see Table 1; LR-EI-MS *m/z* 250, 232, 217, 199, 175, 123, 91, 77, 55; HR-EI-MS *m/z* 250.1561 [M]^+^ (calcd. for C_15_H_22_O_3_, 250.1563).

### 3.4. Cytotoxic Assay

The in vitro cytotoxicities of **1**–**4** were determined by means of the colorimetric 3-(4,5-dimethylthiazol-2-yl)-2,5-diphenyl-2*H*-tetrazolium bromide (MTT) assay. The tested human cancer cell lines were seeded in 96-well plates at a density of 3 × 10^7^ cells/L, and the compounds were added at various concentrations (0.125–50 mg/L). After 48 h, MTT was added to the culture medium at a final concentration of 0.5 mg/mL, and the plates were incubated for 4 h at 37 °C. The supernatant was removed. The formazan crystals were dissolved in DMSO (150 µL) with gentle shaking at room temperature. The absorbance at 570 nm was recorded with a microplate reader (Bio-Rad, Hercules, CA, USA), and the data were analyzed with the SPSS 13.0 software package. Hirsutanol A—a potent anticancer agent isolated from marine fungal metabolites—was used as a positive control, and its cytotoxicities against the tested cancer cell lines are shown in Table 4.

## 4. Conclusions

The marine fungus *Chondrostereum* sp. was cultured in PD medium and afforded three new hirsutane-type sesquiterpenoids, chondrosterins K–M (**1**–**3**), and the known compound anhydroarthrosporone (**4**). These results further indicated that the metabolites produced by *Chondrostereum* sp. in GPY [12,13] medium were different from those in PD [10,11] medium. By altering the fermentation conditions (e.g., carbon and nitrogen sources, inorganic salts), *Chondrostereum* sp. can produce highly functionalized hirsutane derivatives with a surprising chemodiversity. Furthermore, the metabolites isolation work based on ^1^H NMR screening seems to effectively obtain the novel hirsutane-type compounds.

## Figures and Tables

**Table 1:** ^13^C NMR data of compounds **1**–**4**, δ in ppm.

Position	1 ^a^	2		3		4	
	In CDCl_3_	In CDCl_3_^a^	In Acetone-*d*_6_^b^	In CDCl_3_^b^	In Acetone-*d*_6_^b^	In CDCl_3_^a^	In Acetone-*d*_6_^b^
1	42.6, CH	184.8, C	184.6	183.7, C	184.7	63.4, CH	63.6
2	186.4, C	59.3, C	59.4	59.1, C	59.0	53.4, C	53.9
3	135.2, C	156.8, C	158.8	52.1, CH	52.8	57.7, CH	58.1
4	210.1, C	75.9, CH	73.4	77.7, CH	76.1	211.6, C	210.8
5	53.3, CH_2_	46.1, CH_2_	48.7	48.5, CH_2_	50.6	122.9, CH	122.8
6	52.6, C	93.2, C	91.4	91.2, C	90.1	190.8, C	192.7
7	77.8, CH	36.4, CH_2_	40.2	40.1, CH_2_	42.0	43.9, CH_2_	44.5
8	50.2, CH	139.1, C	140.2	141.2, C	141.9	92.7, C	92.7
9	41.6, CH_2_	209.7, C	208.3	209.1, C	208.4	55.9, CH_2_	56.2
10	43.2, C	48.7, C	48.9	48.8, CH	49.0	43.3, C	43.7
11	46.1, CH_2_	39.2, CH_2_	39.4	42.5, CH_3_	42.8	41.8, CH_2_	42.3
12	56.1, CH_2_	19.9, CH_3_	18.4	19.4, CH_3_	19.0	20.8, CH_3_	21.1
13	22.6, CH_3_	110.9, CH_2_	107.4	14.5, CH_3_	14.0	9.5, CH_3_	9.7
14	28.5, CH_3_	25.224, CH_3_	25.5	25.6, CH_3_	25.8	30.2, CH_3_	30.5
15	26.8, CH_3_	25.211, CH_3_	25.4	25.2, CH_3_	25.4	28.1, CH_3_	28.4

^a^ Measured at 100 MHz; ^b^ Measured at 150 MHz.

**Table 2:** ^1^H NMR data of compounds **1** and **2**, δ in ppm, *J* in Hz.

Position	1 ^a^	2	
		In CDCl_3_^a^	In Acetone-*d*_6_^b^
1	3.47, ddd (10.4, 9.6, 9.2)		
4		4.45, dd (4.4, 2.0)	4.32, dddd (7.8, 6.0, 1.8, 1.8)
5	2.35, s	β: 1.87, dd (14.0, 4.4) α: 2.08, dd (14.4, 2.0)	β: 1.85, dd (12.6, 7.8) α: 2.17, dd (12.6, 6.0)
7	4.05, d (9.2)	β: 2.43, dt (16.0, 3.2) α: 2.61, dt (16.0, 2.0)	β: 2.45, ddd (16.8, 3.6, 3.0) α: 2.54, ddd (16.8, 3.6, 2.4)
8	3.16, dddd (9.6, 9.6, 9.2, 9.2)		
9	β: 1.55, ddd (12.8, 9.2, 2.0) α: 1.92, dd (12.8, 9.6)		
11	β: 1.63, dd (12.8, 10.4) α: 1.87, ddd (12.8, 9.2, 2.0)	2.31, dd (3.2, 2.0)	β: 2.27, ddd (18.0, 3.6, 3.0) α: 2.36, ddd (18.0, 3.6, 2.4)
12	β: 4.29, d (13.6) α: 4.34, d (13.6)	1.31, s	1.28, s
13	1.33, s	5.35, s 5.10, s	5.23, d (1.8) 5.07, d (1.8)
14	1.16, s	1.12, s	1.00, s
15	1.01, s	1.06, s	1.07, s
4-OH		2.97, brs	4.16, brs
6-OH		2.97, brs	2.98, brs
7-OH	2.15, brs		
12-OH	2.15, brs		

^a^ Measured at 400 MHz; ^b^ Measured at 600 MHz.

**Table 3:** ^1^H NMR data of compounds **3** and **4**, δ in ppm, *J* in Hz.

Position	3		4	
	In CDCl_3_^b^	In Acetone-*d*_6_^b^	In CDCl_3_^a^	In Acetone-*d*_6_^b^
1			2.38, dd (10.5, 8.4)	2.42, dd (10.2, 9.0)
3	1.93, dq (6.4, 7.2)	1.76, dq (9.6, 7.2)	2.32, q (7.2)	2.27, q (7.2)
4	3.63, dd (6.4, 5.6, 5.6)	3.40, ddd (10.2, 9.6, 6.6)		
5	β: 1.91, dd (13.2, 5.6) α: 2.28, dd (13.2, 5.6)	β: 1.82, dd (12.6, 10.2) α: 2.24, dd (12.6, 6.6)	5.82, d (1.8)	5.69, d (1.8)
7	β: 2.45, d (10.0) α: 2.66, d (10.0)	β: 2.45, d (10.0) α: 2.66, d (10.0)	β: 2.71, dd (15.6, 1.8) α: 2.79, d (15.6)	β: 2.75, d (15.6) α: 2.81, dd (15.6, 1.8)
9			β: 1.66, d (13.8) α: 1.86, dd (13.8, 1.2)	β: 1.70, d (13.8) α: 1.85, dd (13.8, 1.2)
11	β: 2.38, d (10.0) α: 2.44, d (10.0)	β: 2.39, d (10.0) α: 2.41, d (10.0)	β: 1.46, dd (12.9, 10.5) α: 1.70, ddd (12.9, 8.4, 1.2)	β: 1.51, dd (12.6, 10.2) α: 1.67, ddd (12.6, 9.0, 1.2)
12	1.21, s	1.16, s	0.91, s	0.92, s
13	1.06, d (7.2)	1.06, d (7.2)	1.08, d (7.2)	1.00, d (7.2)
14	1.15, s	1.08, s	1.11, s	1.08, s
15	1.12, s	1.03, s	1.20, s	1.19, s
4-OH	2.15, brs	4.17, brs		
6-OH	2.53, brs	4.07, brs		
8-OH			1.98, brs	3.93, brs

^a^ Measured at 400 MHz; ^b^ Measured at 600 MHz.

**Table 4:** Cytotoxicities of compounds **1**–**3**, IC_50_ (μM)

Cancer Cell Lines	1	2	3	Hirsutanol A
CNE1	17.66	33.55	42.00	10.08
CNE2	12.03	22.50	44.08	12.72
HONE1	22.06	34.60	46.11	17.40
SUNE1	16.44	30.40	58.83	3.50
A549	23.51	29.67	49.58	11.96
GLC82	18.08	37.47	55.90	10.11
HL7702	22.14	34.26	56.40	9.76

## Supplementary Materials

The MS and NMR data of compounds **1**–**4** are available online at http://www.mdpi.com/1660-3397/14/9/157/s1. Figure S1: HR-EI-MS spectrum of chondrosterin K (**1**), Figure S2: ^1^H NMR spectrum of chondrosterin K (**1**) in CDCl_3_ (400 MHz), Figure S3: ^13^C NMR spectrum of chondrosterin K (**1**) in CDCl_3_ (100 MHz), Figure S4: HMQC spectrum of chondrosterin K (**1**), Figure S5: ^1^H–^1^H COSY spectrum of chondrosterin K (**1**), Figure S6: HMBC spectrum of chondrosterin K (**1**), Figure S7: NOESY spectrum of chondrosterin K (**1**), Figure S8: HR-EI-MS spectrum of chondrosterin L (**2**), Figure S9: ^1^H NMR spectrum of chondrosterin L (**2**) in CDCl_3_ (400 MHz), Figure S10: ^13^C NMR spectrum of chondrosterin L (**2**) in CDCl_3_ (100 MHz), Figure S11: HMQC spectrum of chondrosterin L (**2**), Figure S12: ^1^H–^1^H COSY spectrum of chondrosterin L (**2**), Figure S13: HMBC spectrum of chondrosterin L (**2**), Figure S14: NOESY spectrum of chondrosterin L (**2**), Figure S15: ^1^H NMR spectrum of chondrosterin L (**2**) in Acetone-*d*_6_ (600 MHz), Figure S16: ^13^C NMR spectrum of chondrosterin L (**2**) in Acetone-*d*_6_ (150 MHz), Figure S17: HR-EI-MS spectrum of chondrosterin M (**3**), Figure S18: ^1^H NMR spectrum of chondrosterin M (**3**) in CDCl_3_ (600 MHz), Figure S19: ^13^C NMR spectrum of chondrosterin M (**3**) in CDCl_3_ (150 MHz), Figure S20: HMQC spectrum of chondrosterin M (**3**), Figure S21: ^1^H–^1^H COSY spectrum of chondrosterin M (**3**), Figure S22: HMBC spectrum of chondrosterin M (**3**), Figure S23: NOESY spectrum of chondrosterin M (**3**), Figure S24: ^1^H NMR spectrum of chondrosterin M (**3**) in Acetone-*d*_6_ (600 MHz), Figure S25: ^13^C NMR spectrum of chondrosterin M (**3**) in Acetone-*d*_6_ (150 MHz), Figure S26: HR-EI-MS spectrum of anhydroarthrosporone (**4**), Figure S27: ^1^H NMR spectrum of anhydroarthrosporone (**4**) in CDCl_3_ (400 MHz), Figure S28: ^13^C NMR spectrum of anhydroarthrosporone (**4**) in CDCl_3_ (100 MHz), Figure S29: HMQC spectrum of anhydroarthrosporone (**4**), Figure S30: ^1^H–^1^H COSY spectrum of anhydroarthrosporone (**4**), Figure S31: HMBC spectrum of anhydroarthrosporone (**4**), Figure S32: NOESY spectrum of anhydroarthrosporone (**4**), Figure S33: ^1^H NMR spectrum of anhydroarthrosporone (**4**) in Acetone-*d*_6_ (600 MHz), Figure S34: ^13^C NMR spectrum of anhydroarthrosporone (**4**) in Acetone-*d*_6_ (150 MHz).
