# Supplementary material for: Additional New Cytotoxic Triquinane-Type Sesquiterpenoids Chondrosterins K–M from the Marine Fungus Chondrostereum sp
Source: Mar Drugs. 2016 Aug 26;14(9):157. doi: 10.3390/md14090157 (PMC5039528; doi:10.3390/md14090157)
Supplement: Supplementary file 1 [file marinedrugs-14-00157-s001.pdf]

Supplementary Materials: Additional New Cytotoxic Triquinane-Type Sesquiterpenoids Chondrosterins K–M from the Marine Fungus *Chondrostereum* sp.

Lei Huang, Wen-Jian Lan, Rong Deng, Gong-Kan Feng, Qing-Yan Xu, Zhi-Yu Hu, Xiao-Feng Zhu and Hou-Jin Li

List of Supporting Information **Page**

Figure S1. HR-EI-MS spectrum of chondrosterin K (**1**) S2
Figure S2. ^{1}H NMR spectrum of chondrosterin K (**1**) in $CDCl_3$, 400 MHz S2
Figure S3. ^{13}C NMR spectrum of chondrosterin K (**1**) in $CDCl_3$, 100 MHz S3
Figure S4. HMQC spectrum of chondrosterin K (**1**) S3
Figure S5. ^{1}H–^{1}H COSY spectrum of chondrosterin K (**1**) S4
Figure S6. HMBC spectrum of chondrosterin K (**1**) S4
Figure S7. NOESY spectrum of chondrosterin K (**1**) S5
Figure S8. HR-EI-MS spectrum of chondrosterin L (**2**) S5
Figure S9. ^{1}H NMR spectrum of chondrosterin L (**2**) in $CDCl_3$, 400 MHz S6
Figure S10. ^{13}C NMR spectrum of chondrosterin L (**2**) in $CDCl_3$, 100 MHz S6
Figure S11. HMQC spectrum of chondrosterin L (**2**) S7
Figure S12. ^{1}H–^{1}H COSY spectrum of chondrosterin L (**2**) S7
Figure S13. HMBC spectrum of chondrosterin L (**2**) S8
Figure S14. NOESY spectrum of chondrosterin L (**2**) S8
Figure S15. ^{1}H NMR spectrum of chondrosterin L (**2**) in Acetone-d_6, 600 MHz S9
Figure S16. ^{13}C NMR spectrum of chondrosterin L (**2**) in Acetone-d_6, 150 MHz S9
Figure S17. HR-EI-MS spectrum of chondrosterin M (**3**) S10
Figure S18. ^{1}H NMR spectrum of chondrosterin M (**3**) in $CDCl_3$, 600 MHz S10
Figure S19. ^{13}C NMR spectrum of chondrosterin M (**3**) in $CDCl_3$, 150 MHz S11
Figure S20. HMQC spectrum of chondrosterin M (**3**) S11
Figure S21. ^{1}H–^{1}H COSY spectrum of chondrosterin M (**3**) S12
Figure S22. HMBC spectrum of chondrosterin M (**3**) S12
Figure S23. NOESY spectrum of chondrosterin M (**3**) S13
Figure S24. ^{1}H NMR spectrum of chondrosterin M (**3**) in Acetone-d_6, 600 MHz S13
Figure S25. ^{13}C NMR spectrum of chondrosterin M (**3**) in Acetone-d_6, 150 MHz S14
Figure S26. HR-EI-MS spectrum of anhydroarthrosporone (**4**) S14
Figure S27. ^{1}H NMR spectrum of anhydroarthrosporone (**4**) in $CDCl_3$, 400 MHz S15
Figure S28. ^{13}C NMR spectrum of anhydroarthrosporone (**4**) in $CDCl_3$, 100 MHz S15
Figure S29. HMQC spectrum of anhydroarthrosporone (**4**) S16
Figure S30. ^{1}H–^{1}H COSY spectrum of anhydroarthrosporone (**4**) S16
Figure S31. HMBC spectrum of anhydroarthrosporone (**4**) S17
Figure S32. NOESY spectrum of anhydroarthrosporone (**4**) S17
Figure S33. ^{1}H NMR spectrum of anhydroarthrosporone (**4**) in Acetone-d_6, 600 MHz S18
Figure S34. ^{13}C NMR spectrum of anhydroarthrosporone (**4**) in Acetone-d_6, 150 MHz S18

Mass	Relative Intensity	Theoretical Mass	Delta	Delta [ppm]	DB [mmu]	Composition
232.1456	90.9	232.1458	-0.8	-0.2	6.0	$C_{15}H_{20}O_2$

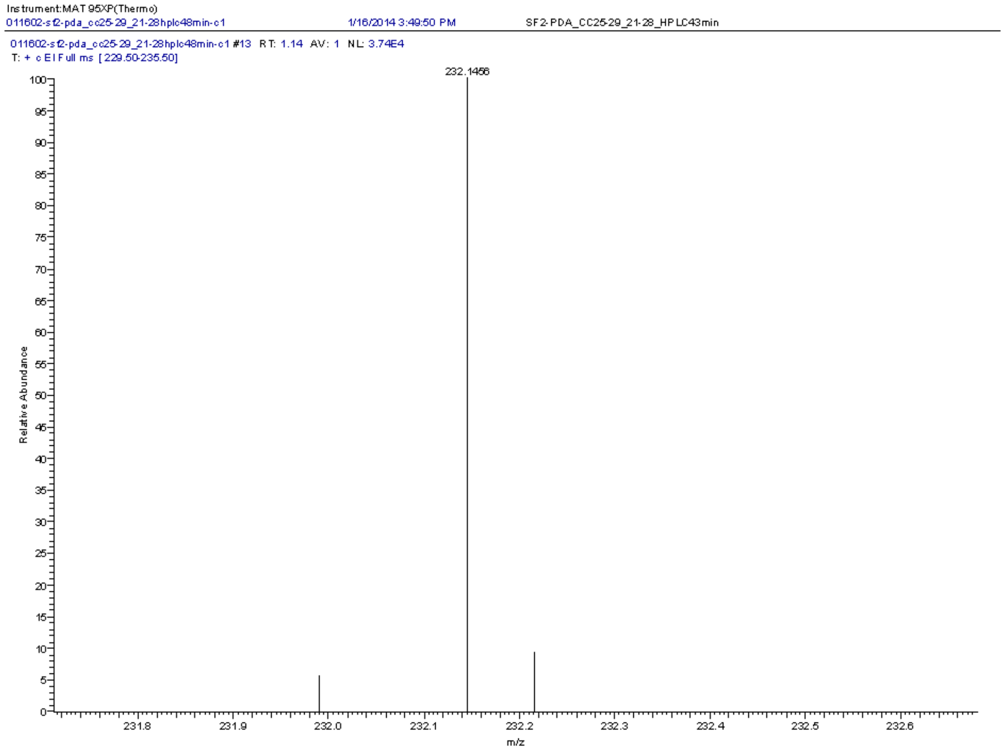

Figure S1. HR-EI-MS spectrum of chondrosterin K (**1**).

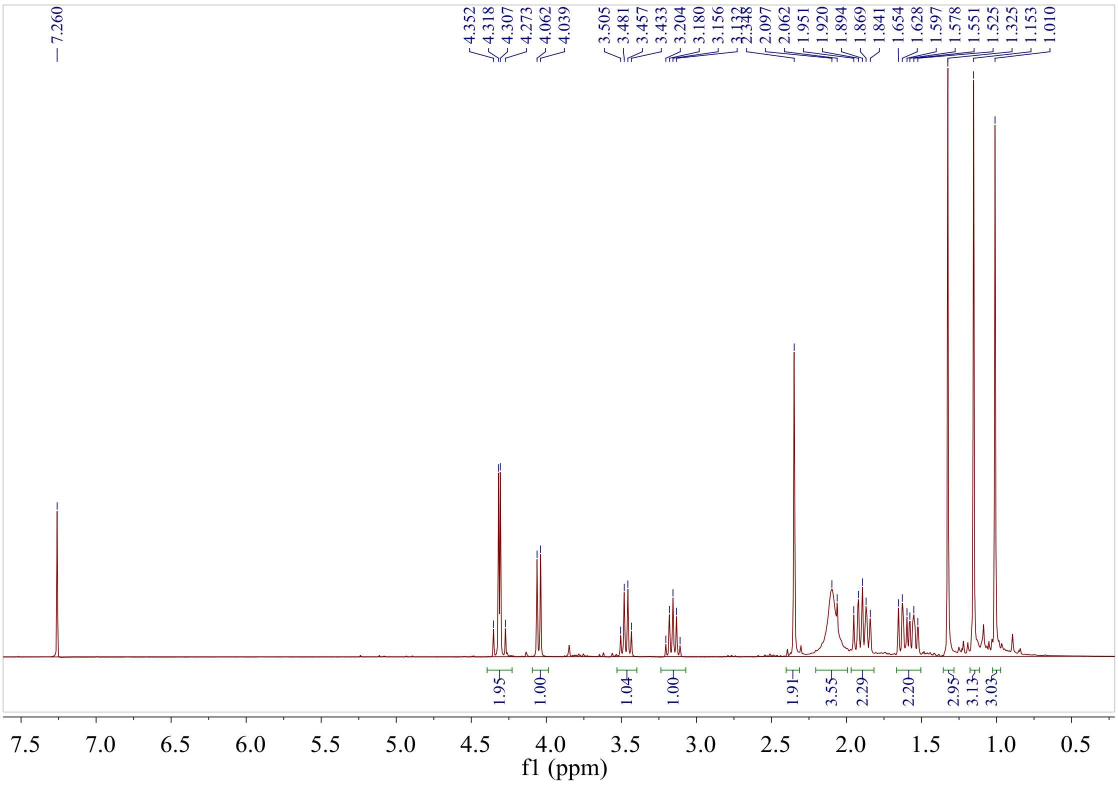

Figure S2. ^1H NMR spectrum of chondrosterin K (**1**) in $CDCl_3$, 400 MHz.

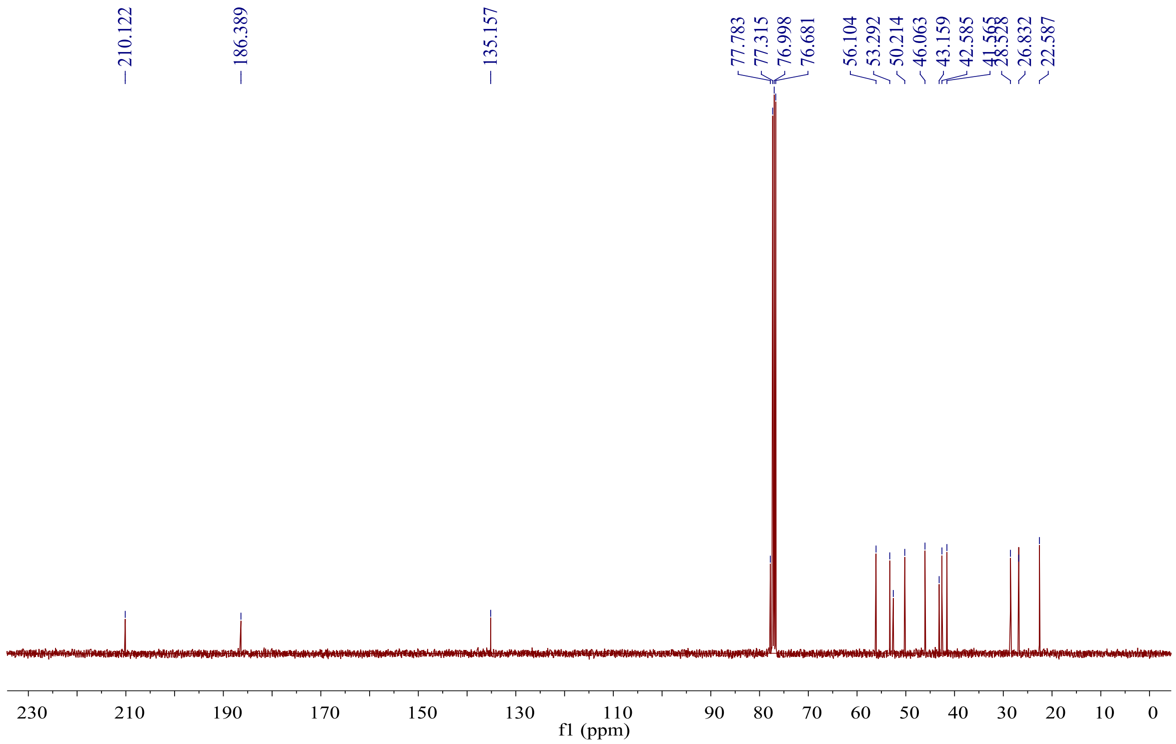

Figure S3. ^{13}C NMR spectrum of chondrosterin K (**1**) in $CDCl_3$, 100 MHz.

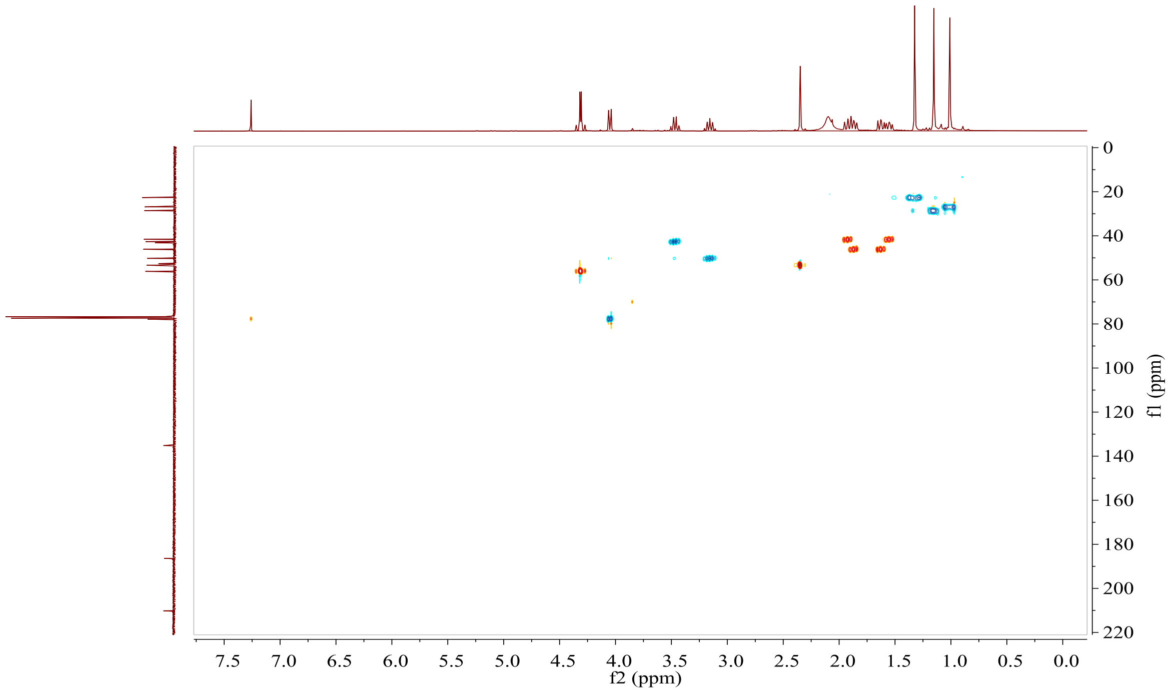

Figure S4. HMQC spectrum of chondrosterin K (**1**).

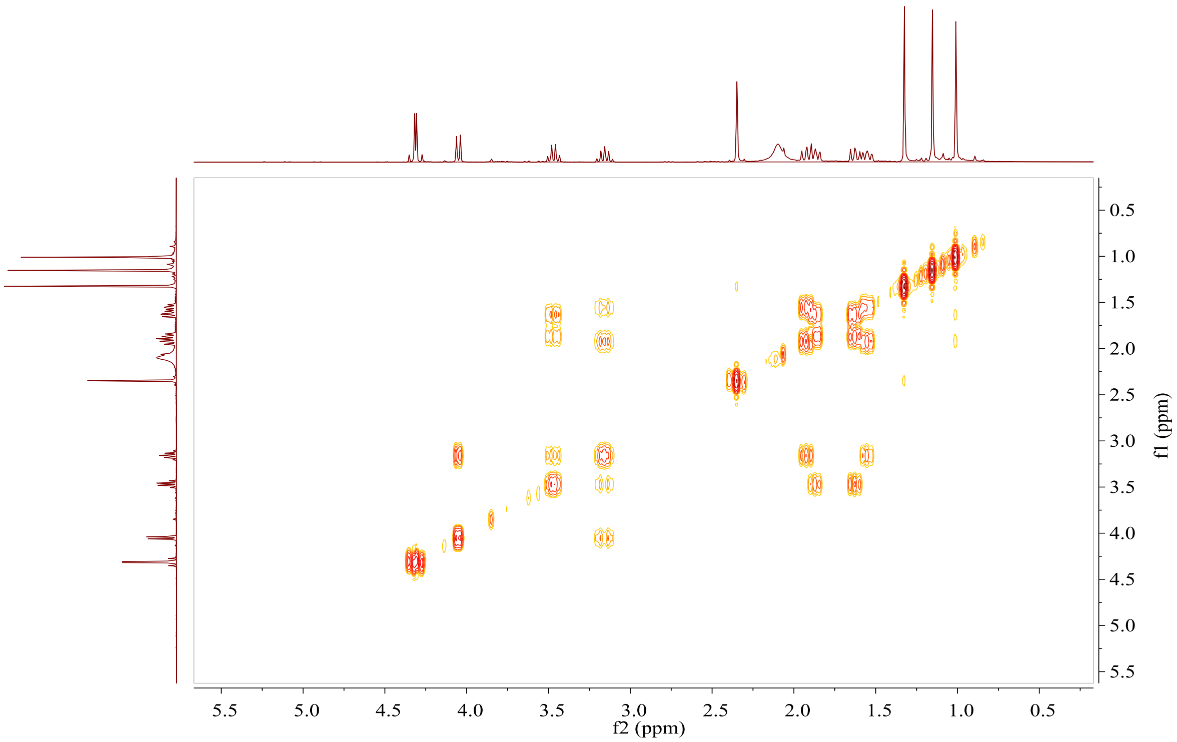

Figure S5. $^1H–^1H$ COSY spectrum of chondrosterin K (**1**).

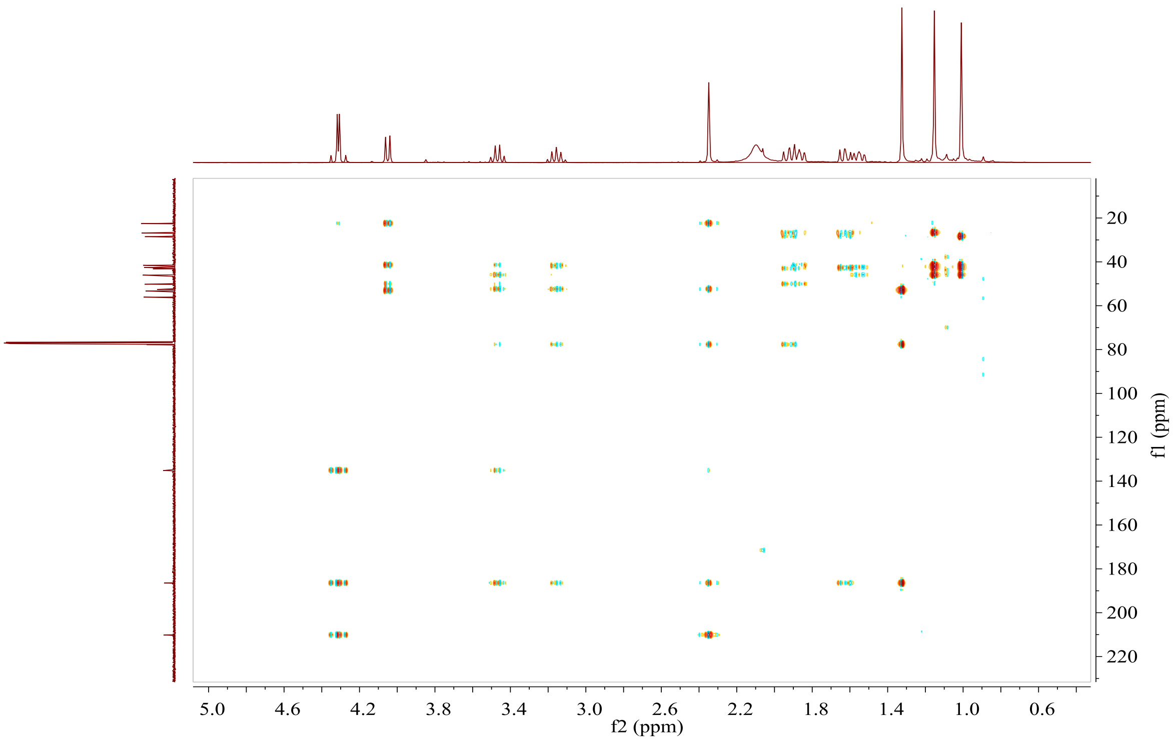

Figure S6. HMBC spectrum of chondrosterin K (**1**).

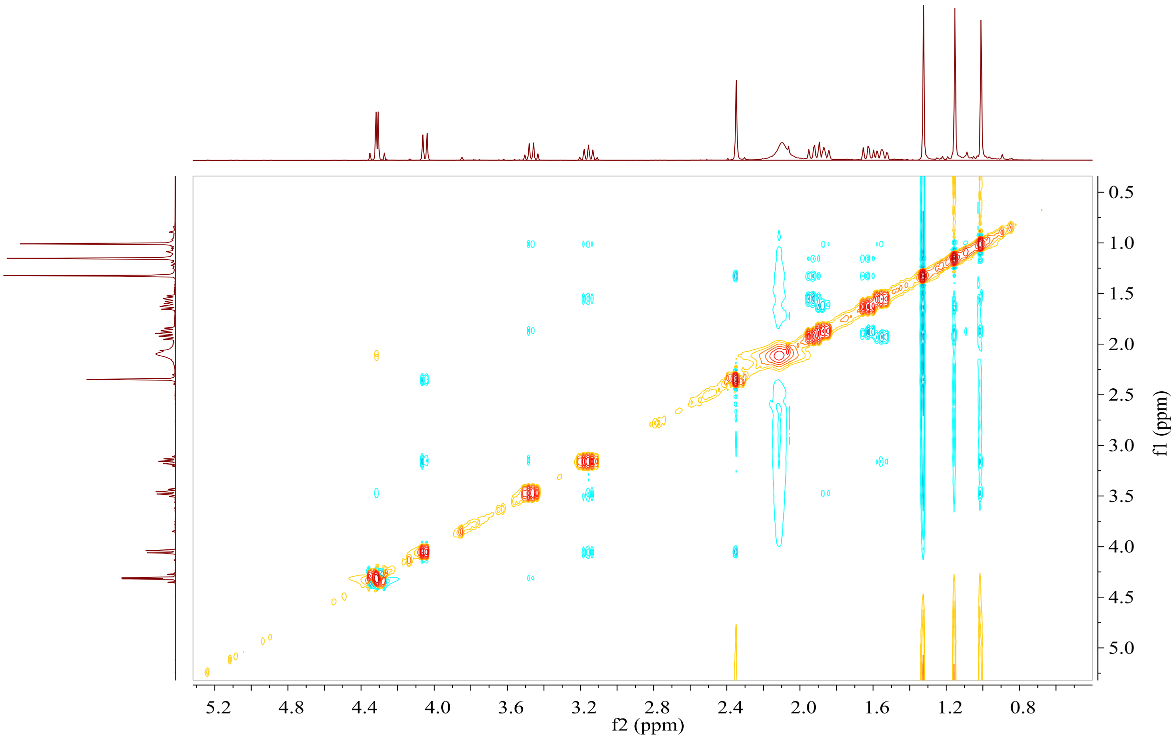

Figure S7. NOESY spectrum of chondrosterin K (**1**).

Mass	Relative Intensity	Theoretical Mass	Delta	Delta [ppm]	RDB [mmu]	Composition
248.1410	100.0	248.1407	1.4	0.3	6.0	C_{15} H_{20} O_3

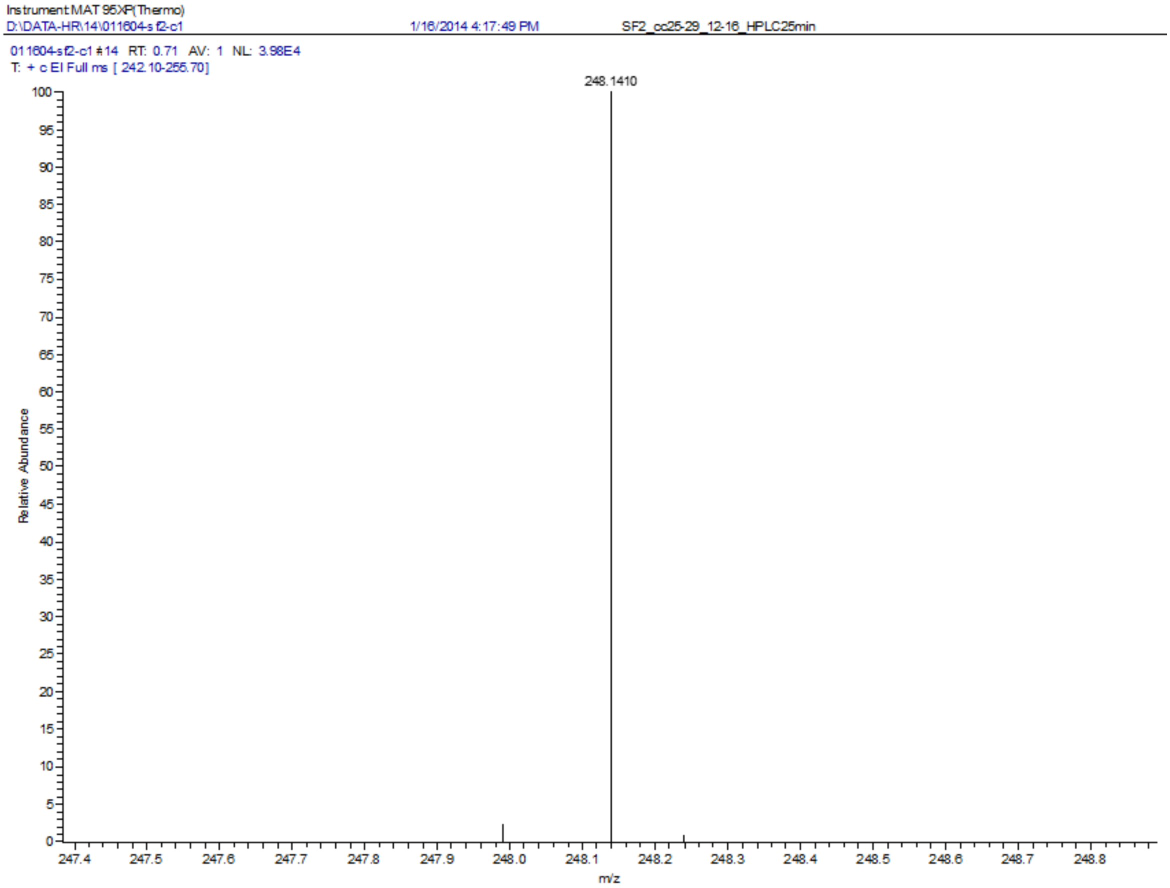

Figure S8. HR-EI-MS spectrum of chondrosterin L (**2**).

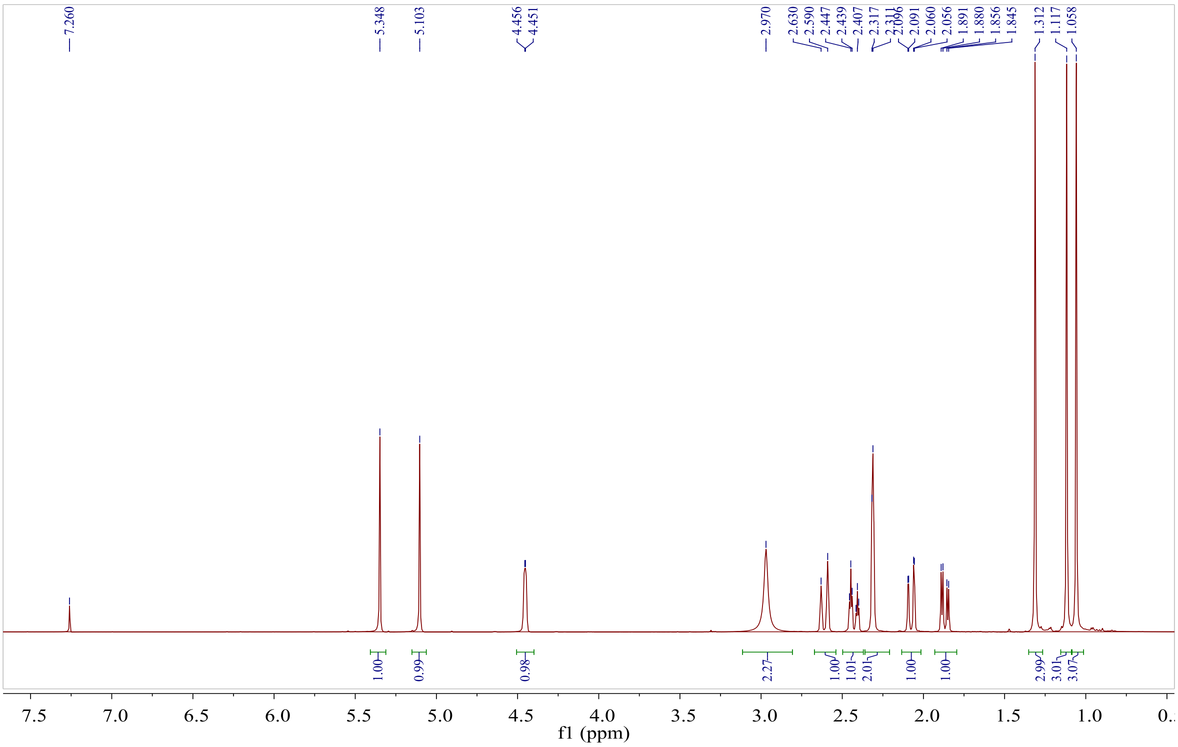

Figure S9. ^{1}H NMR spectrum of chondrosterin L (**2**) in $CDCl_3$, 400 MHz.

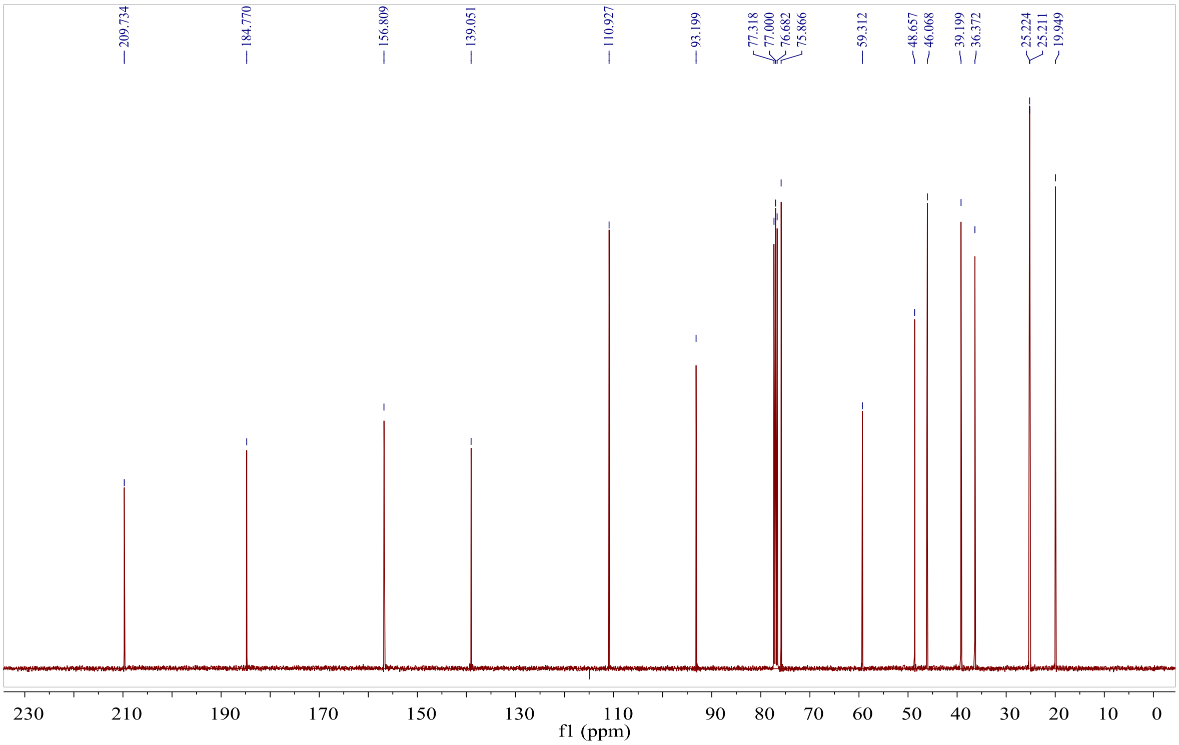

Figure S10. ^{13}C NMR spectrum of chondrosterin L (**2**) in $CDCl_3$, 100 MHz.

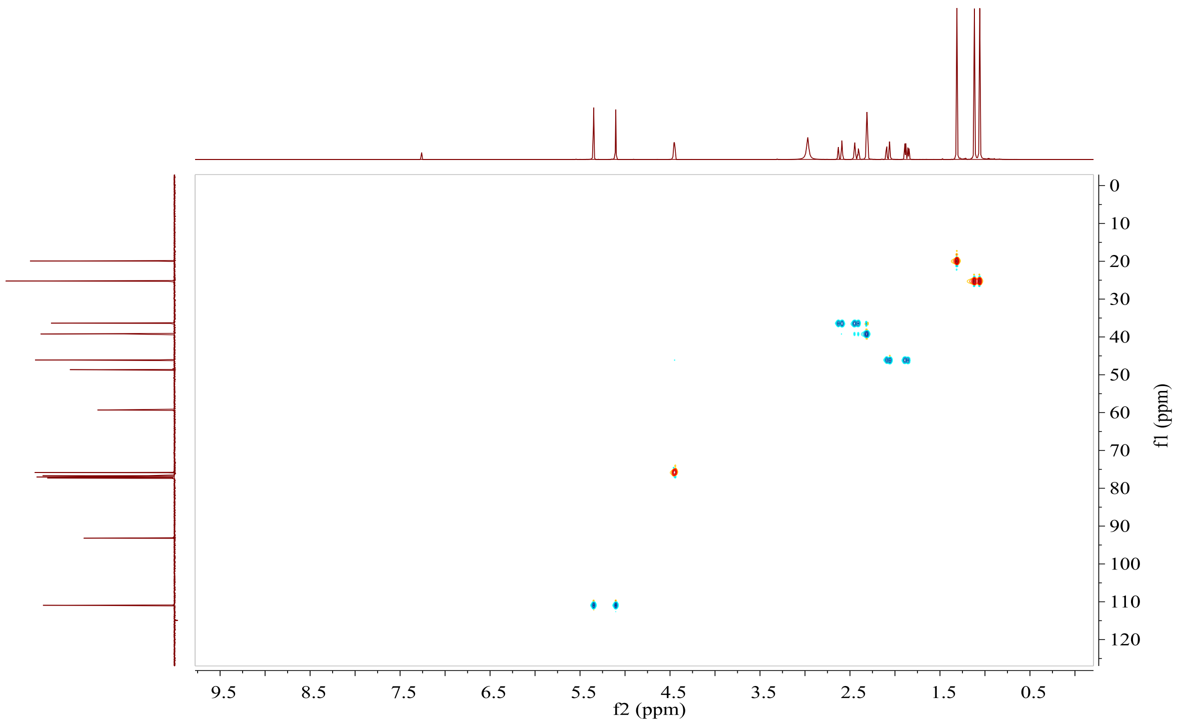

Figure S11. HMQC spectrum of chondrosterin L (**2**).

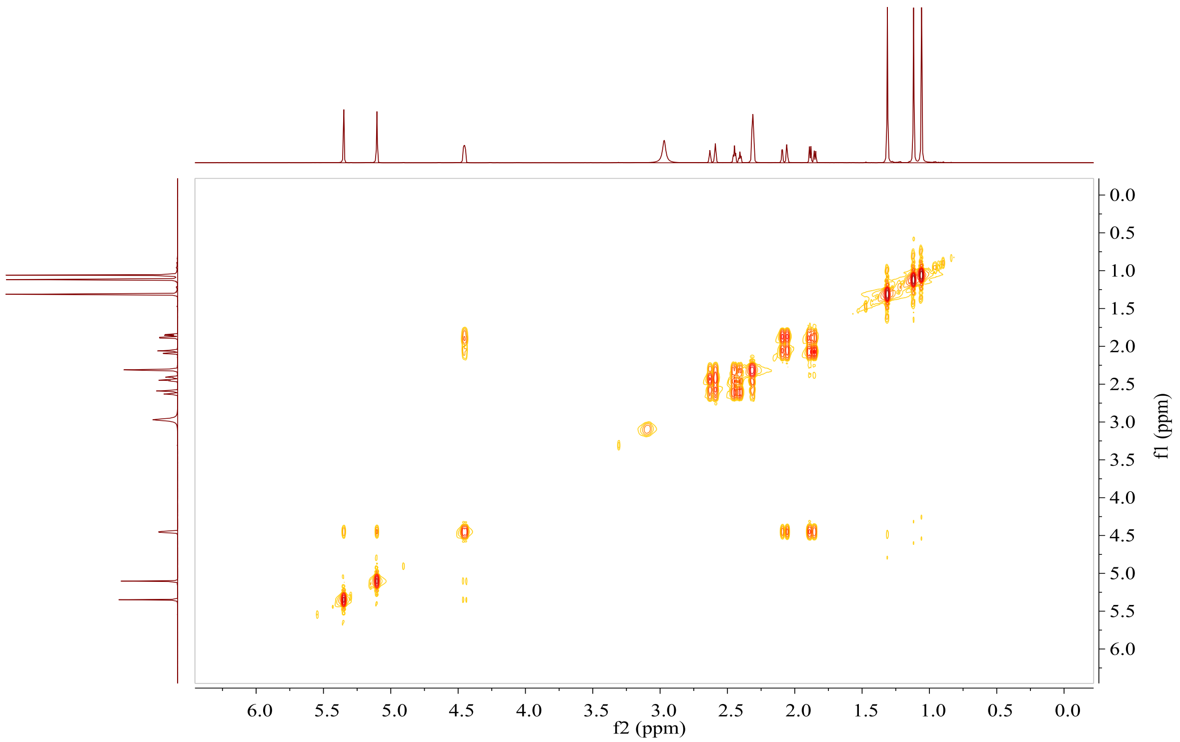

Figure S12. ^{1}H–^{1}H COSY spectrum of chondrosterin L (**2**).

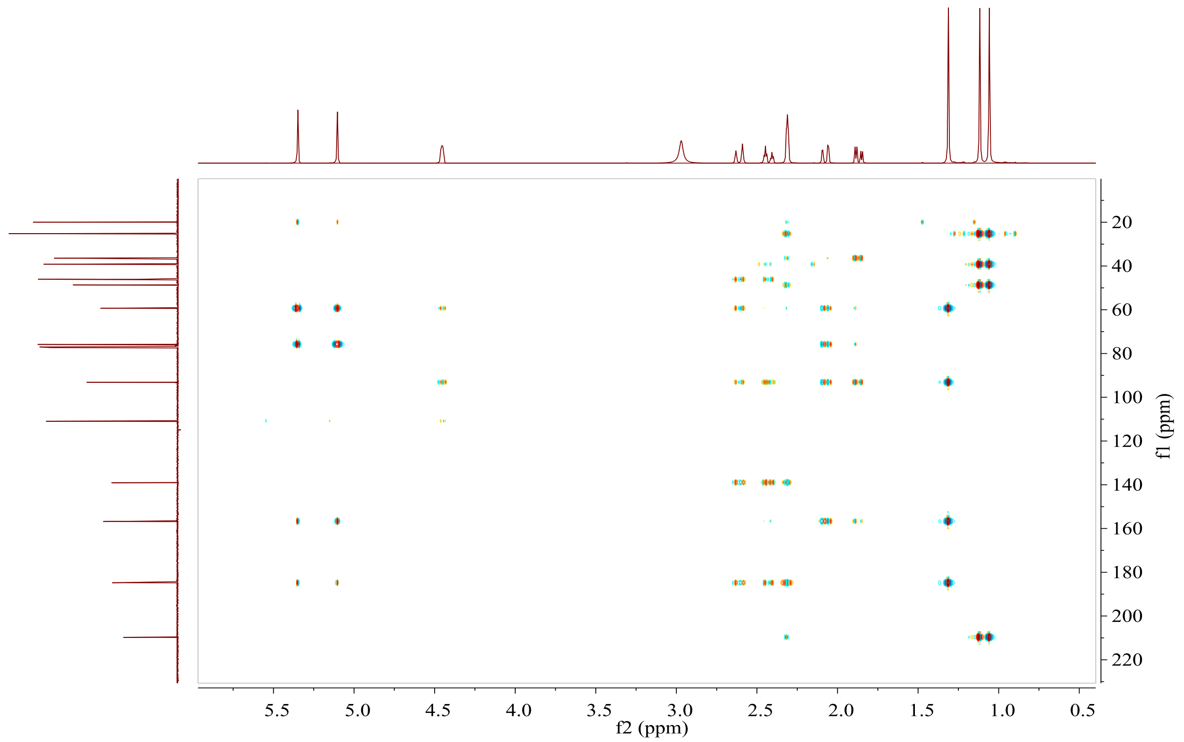

Figure S13. HMBC spectrum of chondrosterin L (**2**).

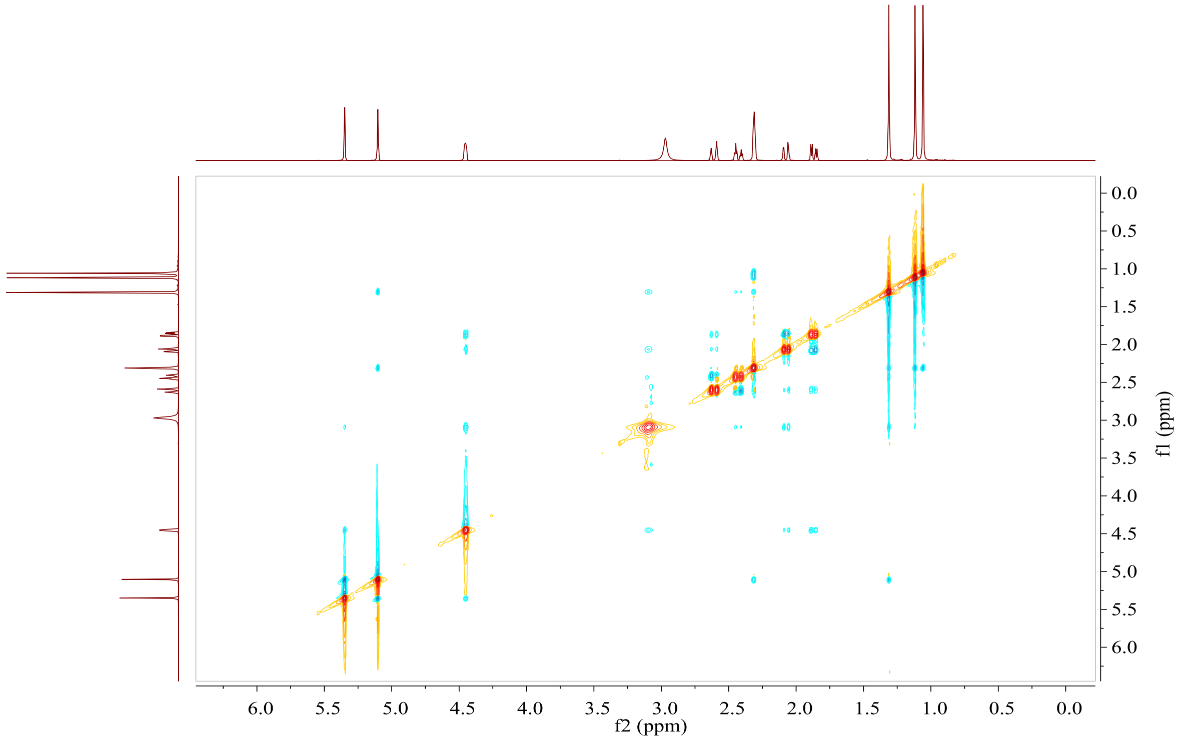

Figure S14. NOESY spectrum of chondrosterin L (**2**).

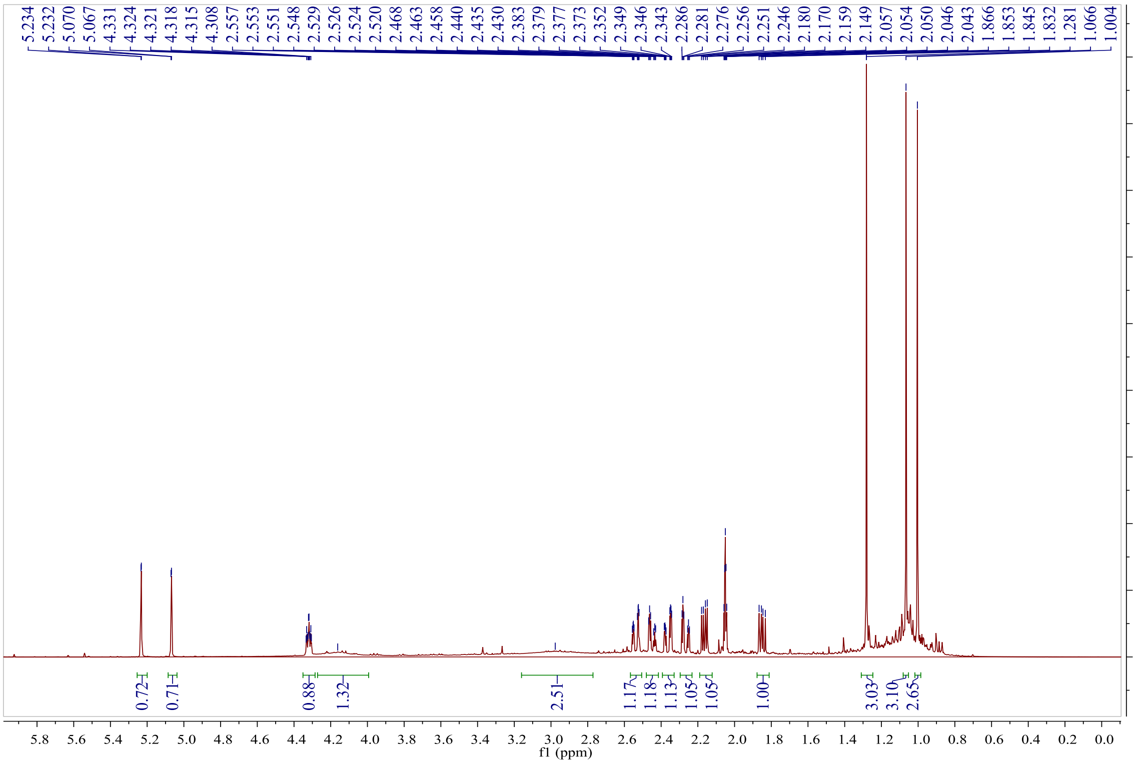

Figure S15. ^{1}H NMR spectrum of chondrosterin L (**2**) in Acetone-*d*6, 600 MHz.

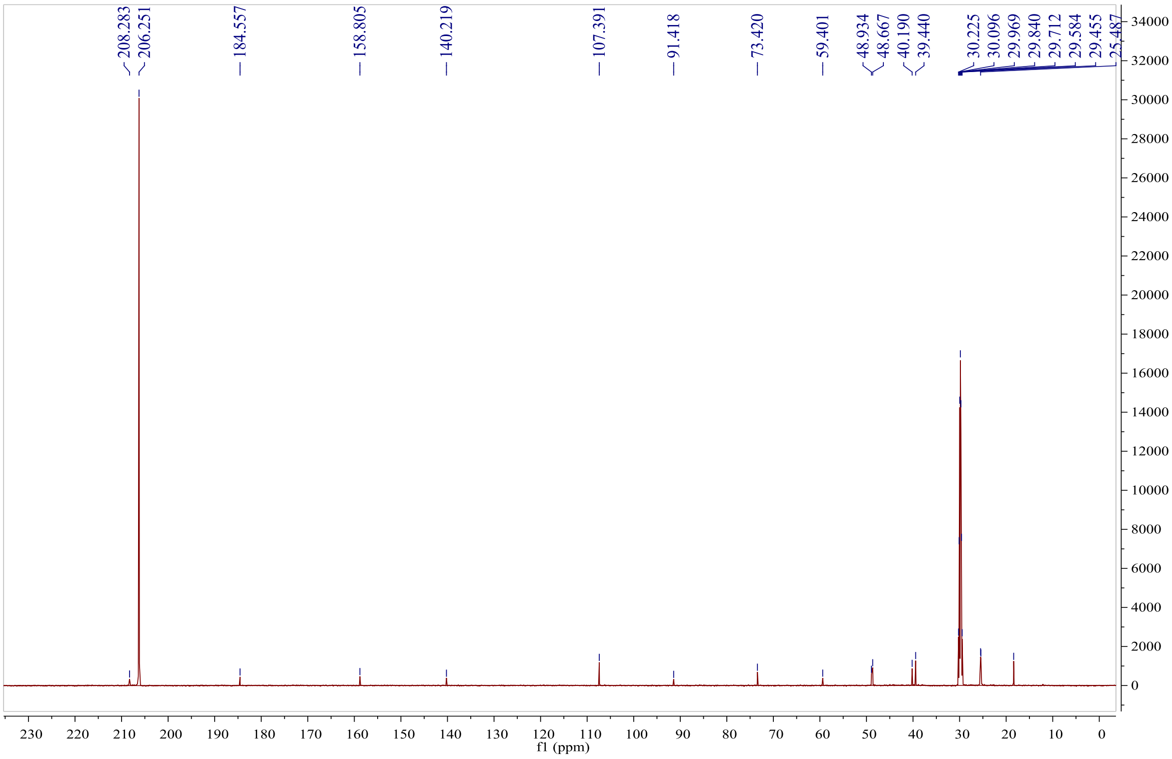

Figure S16. ^{13}C NMR spectrum of chondrosterin L (**2**) in Acetone-*d*6, 150 MHz.

Mass	Relative Intensity	Theoretical Mass	Delta	Delta [ppm]	RDB [mmu]	Composition
250.1561	75.2	250.1563	-0.9	-0.2	5.0	$C_{15}H_{22}O_3$

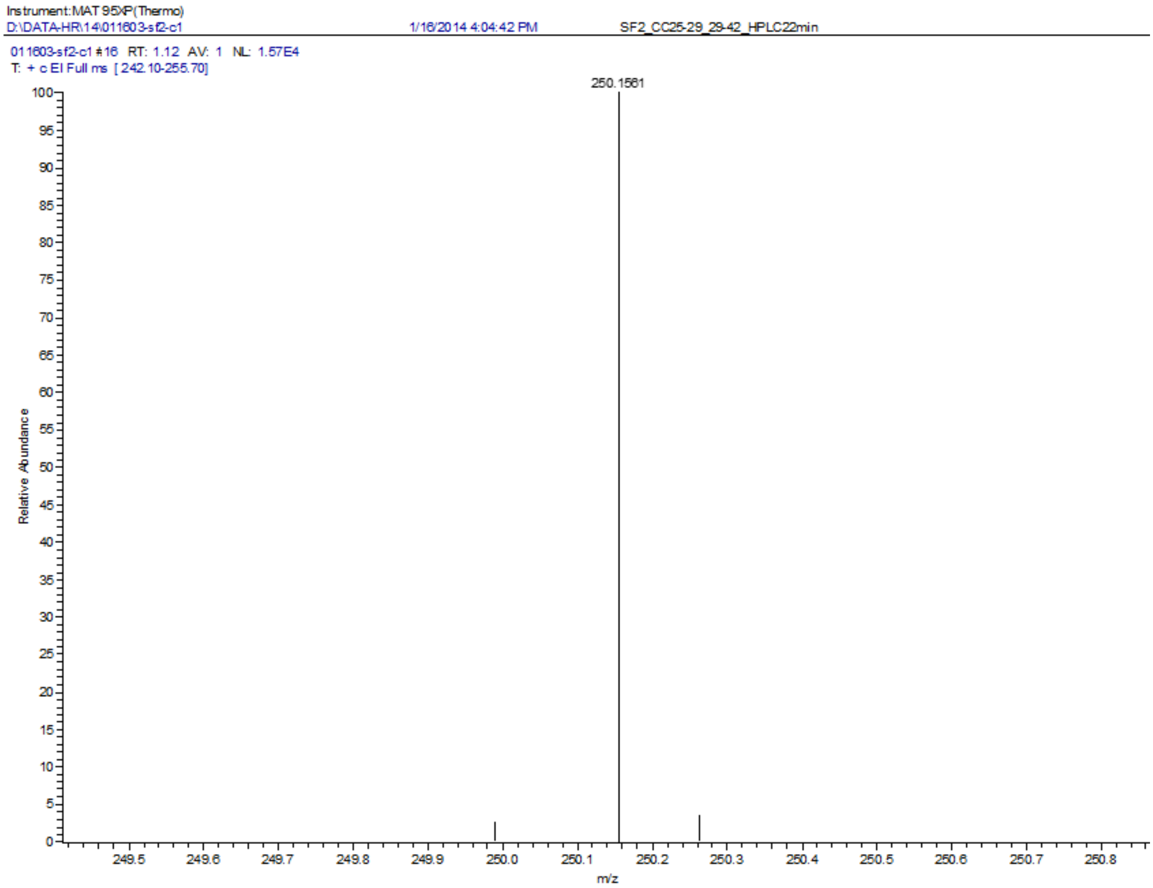

Figure S17. HR-EI-MS spectrum of chondrosterin M (**3**).

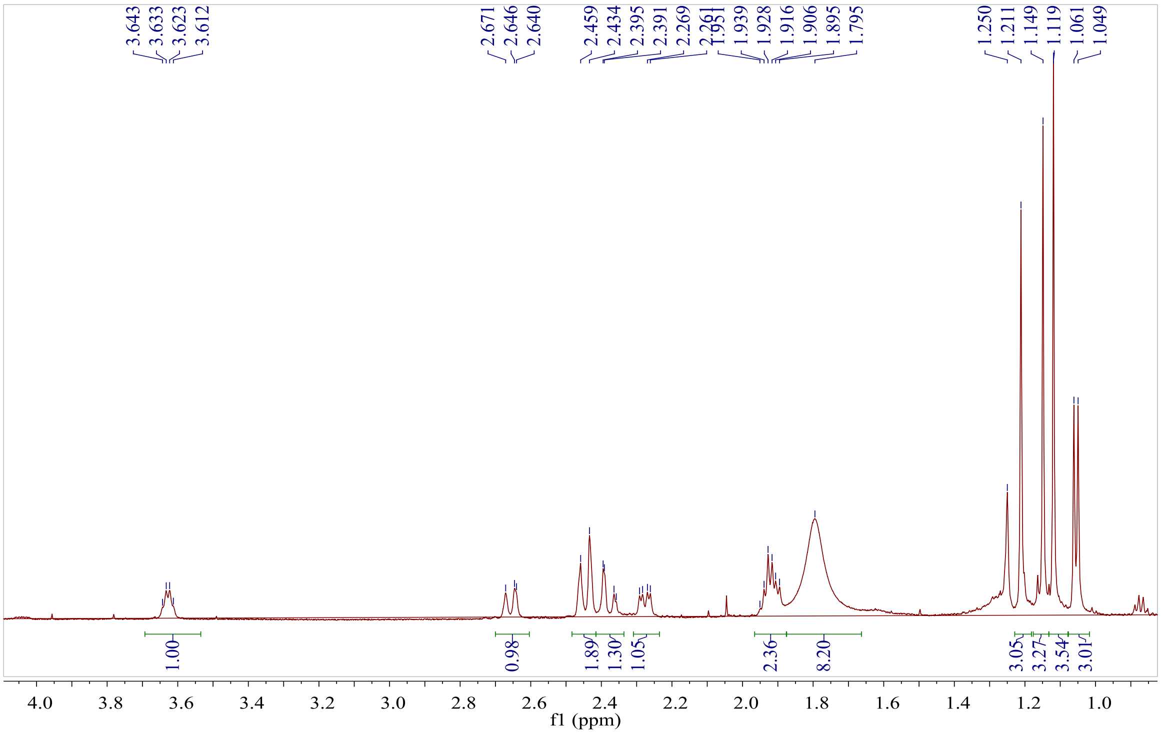

Figure S18. ^{1}H NMR spectrum of chondrosterin M (**3**) in $CDCl_3$, 600 MHz.

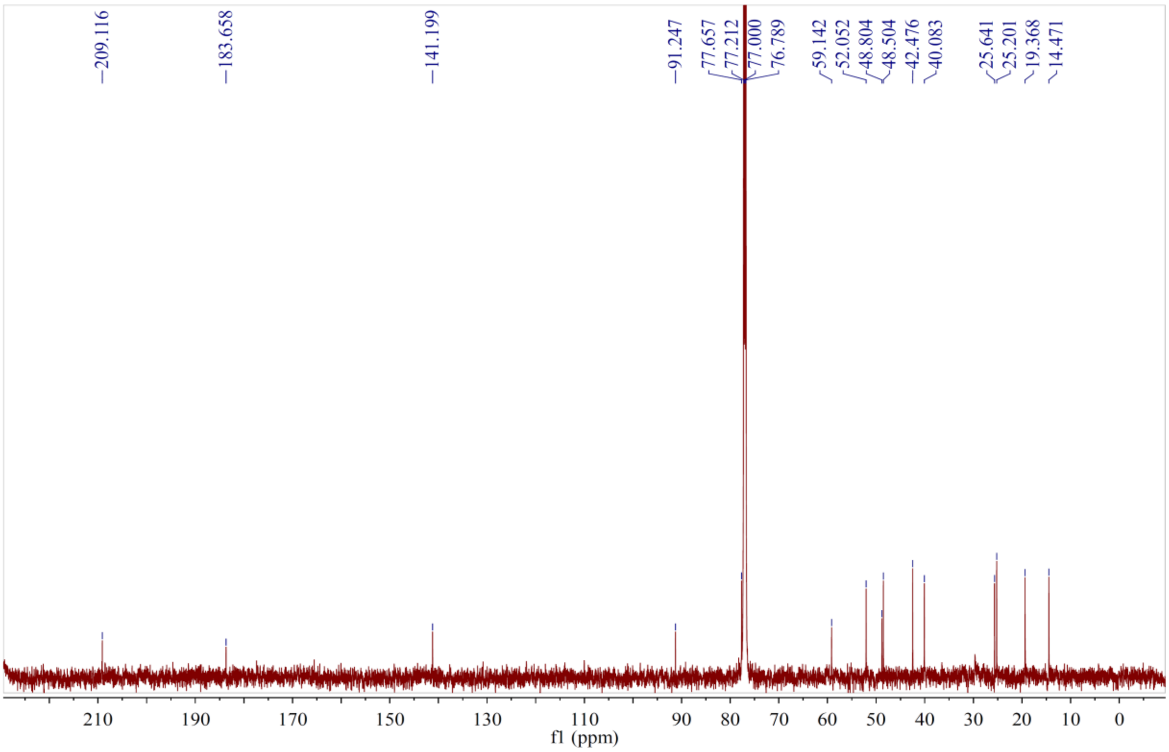

Figure S19. ^{13}C NMR spectrum of chondrosterin M (**3**) in $CDCl_3$, 150 MHz.

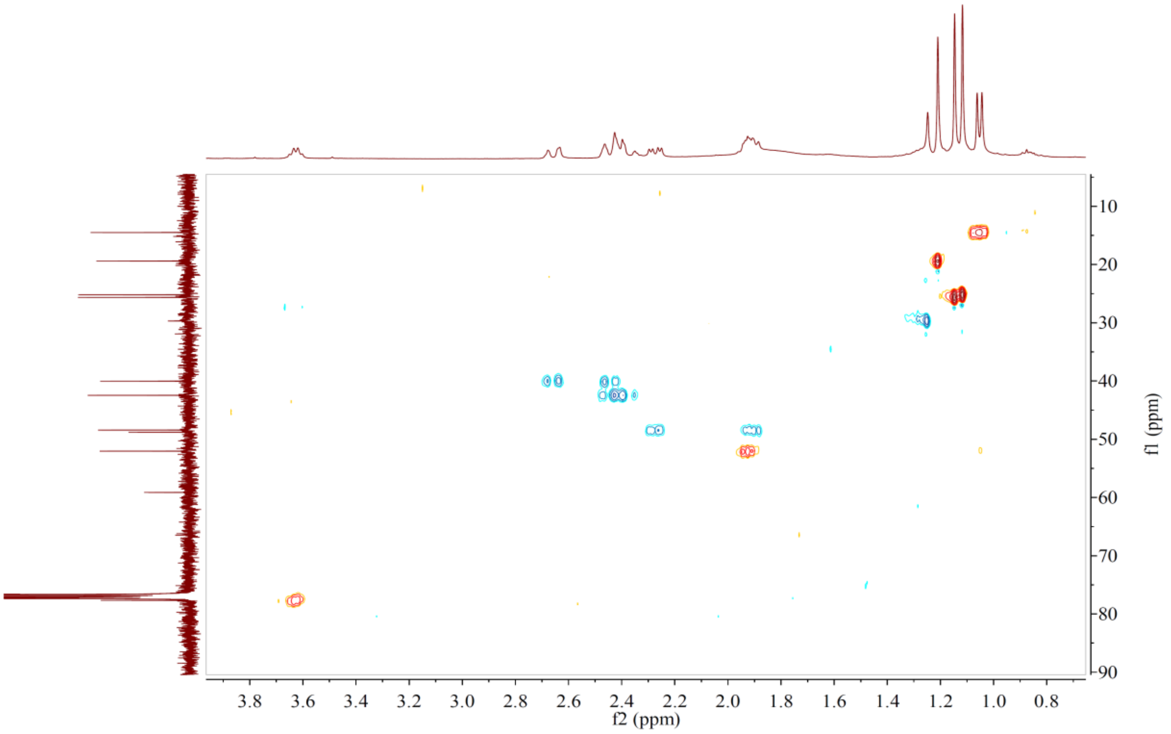

Figure S20. HMQC spectrum of chondrosterin M (**3**).

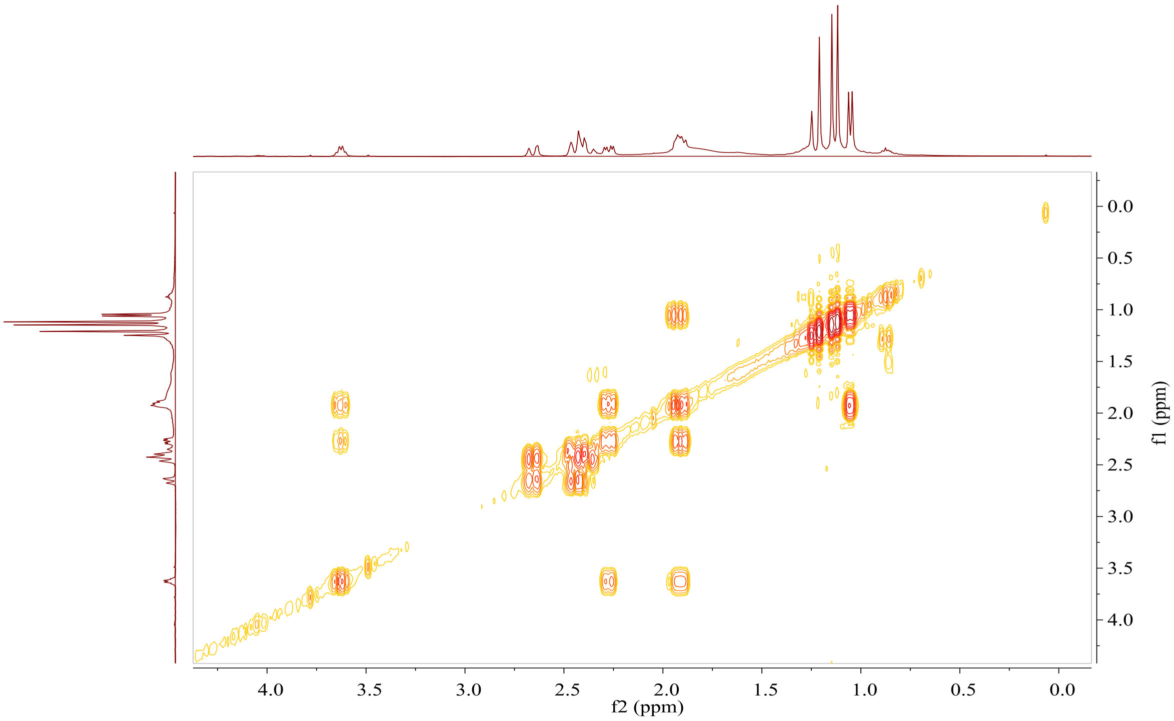

Figure S21. ^{1}H–^{1}H COSY spectrum of chondrosterin M (**3**).

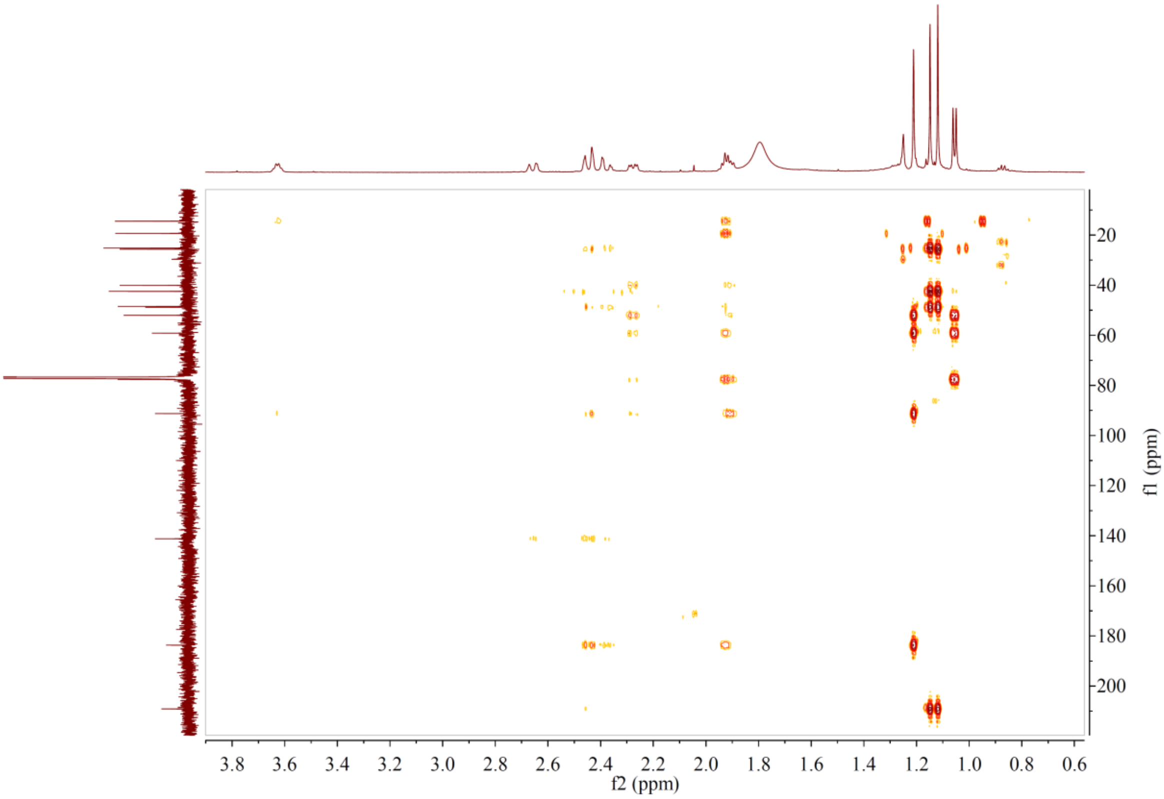

Figure S22. HMBC spectrum of chondrosterin M (**3**).

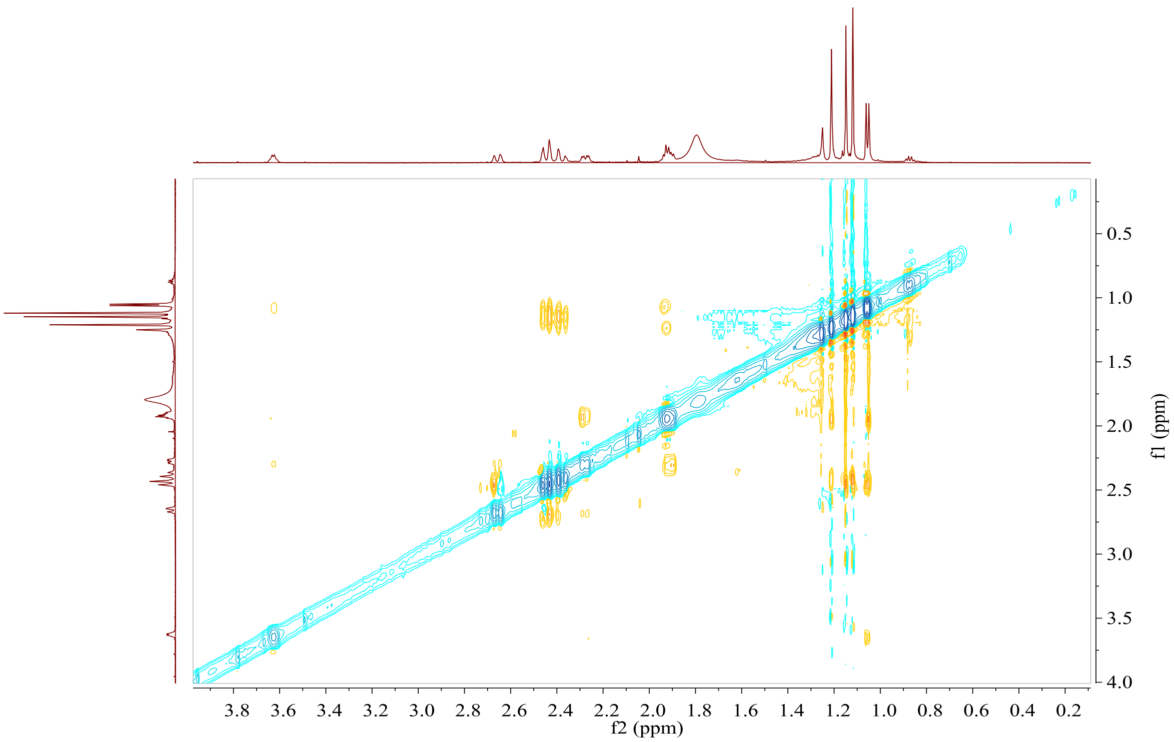

Figure S23. NOESY spectrum of chondrosterin M (**3**).

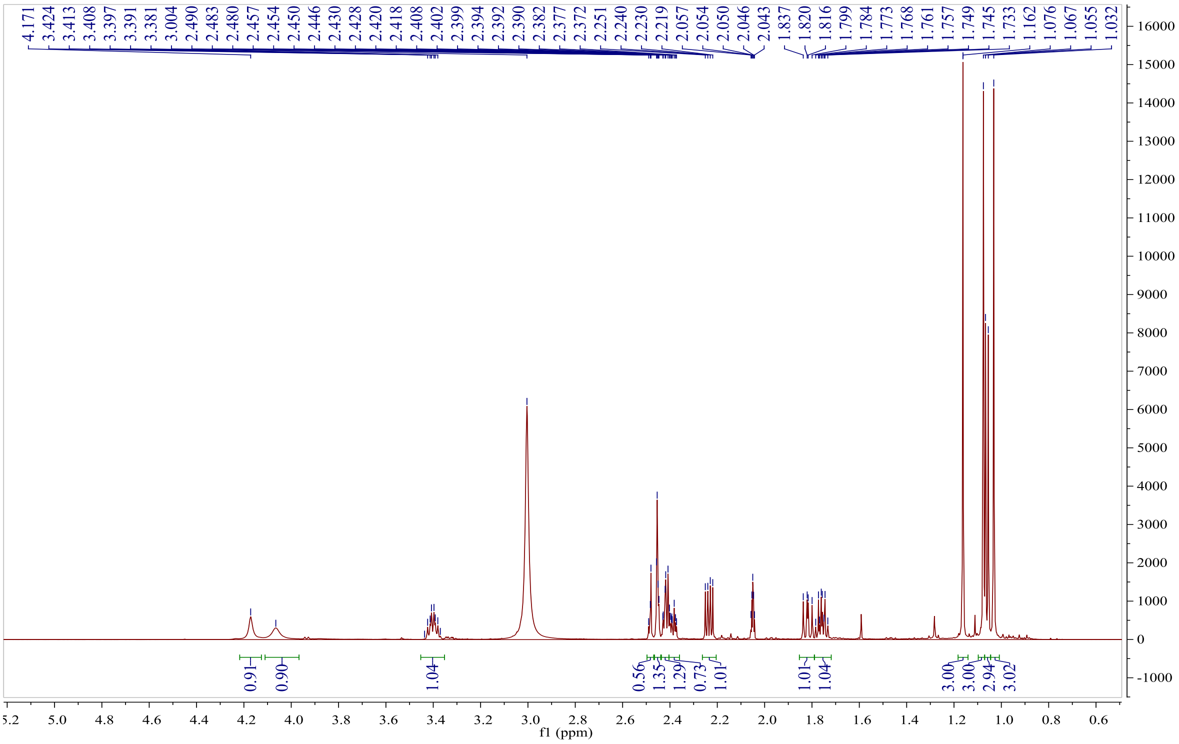

Figure S24. ^{1}H NMR spectrum of chondrosterin M (**3**) in Acetone-d_6, 600 MHz.

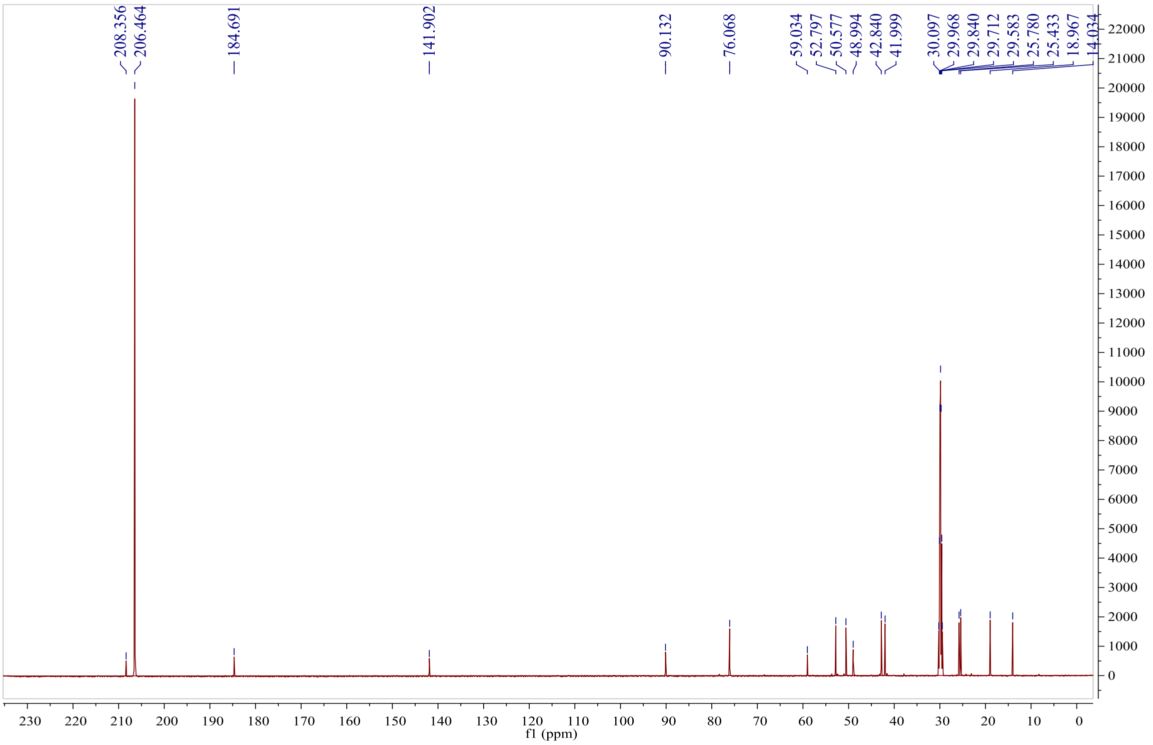

Figure S25. ^{13}C NMR spectrum of chondrosterin M (**3**) in Acetone-*d*6, 150 MHz.

Mass	Relative Intensity	Theoretical Mass	Delta[ppm]	Delta[mmu]	RDB	Composition
234.1613	65.5	234.1614	-0.4	-0.1	5.0	$C_{15}H_{22}O_2$

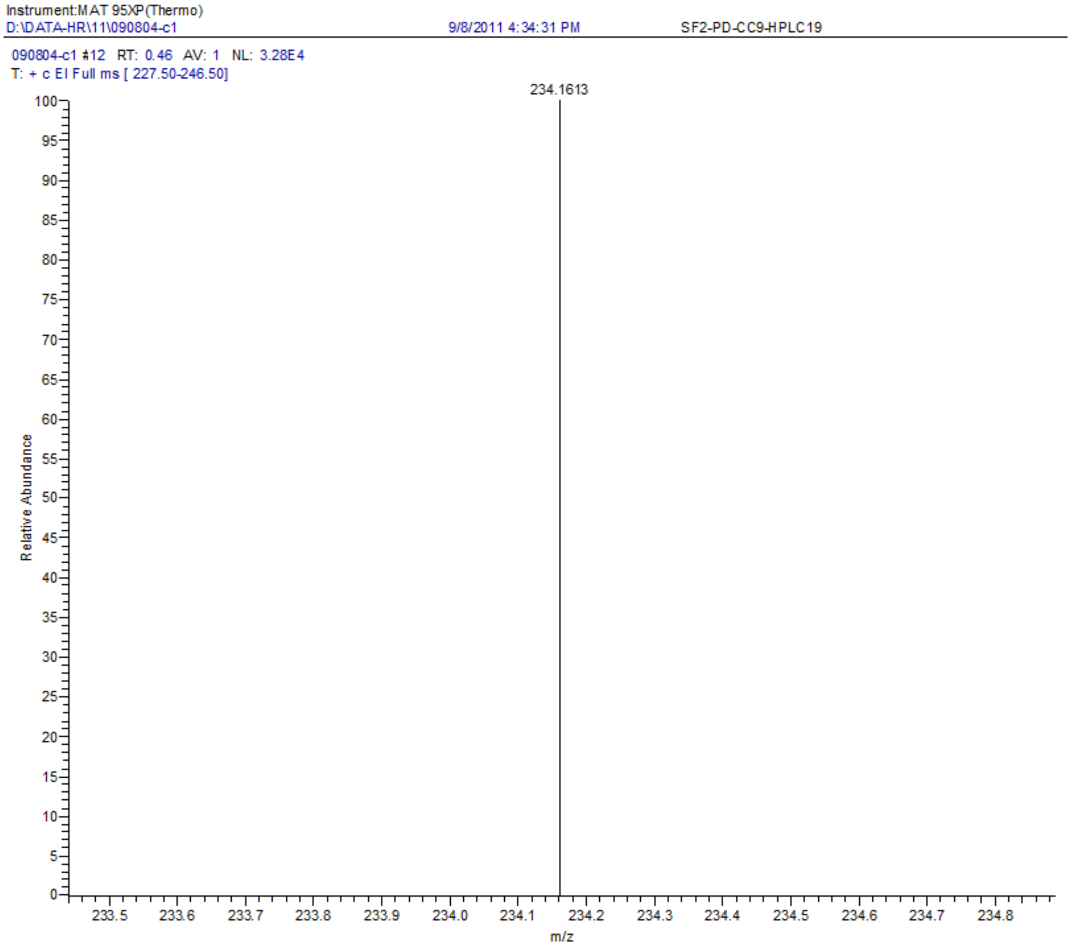

Figure S26. HR-EI-MS spectrum of anhydroarthrosporone (**4**).

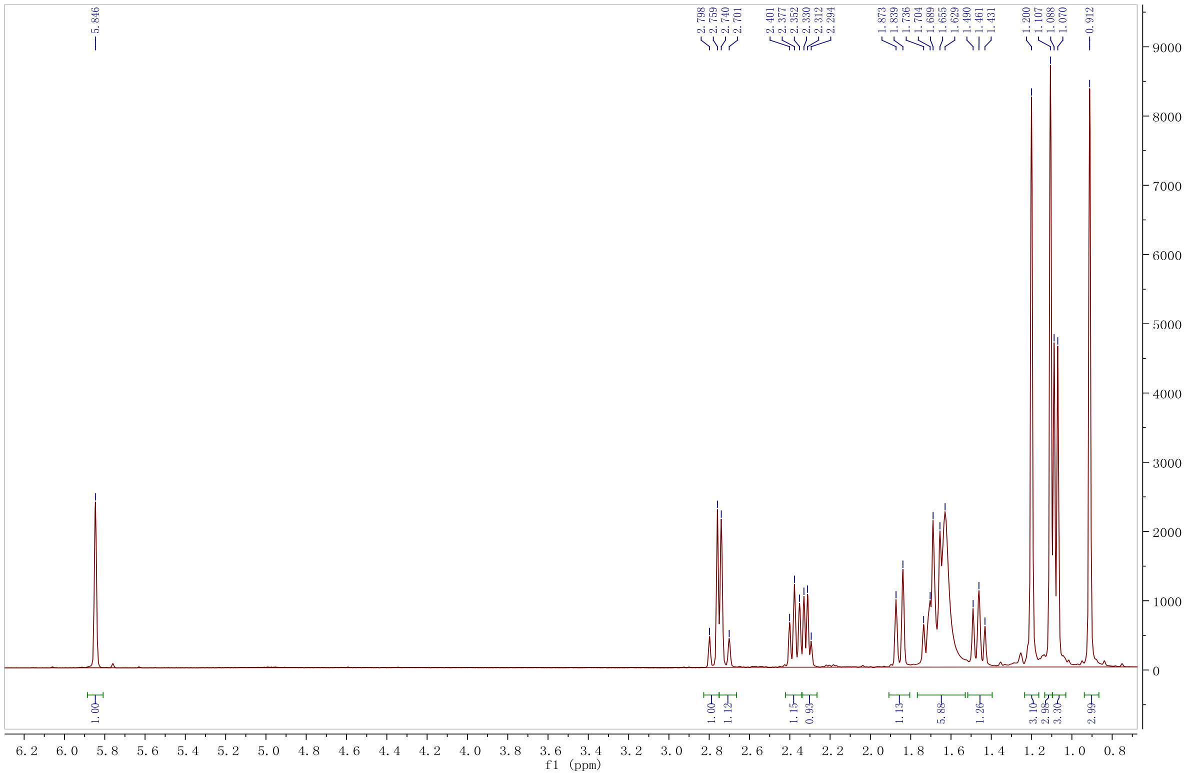

Figure S27. ^{1}H NMR spectrum of anhydroarthrosporone (**4**) in $CDCl_3$, 400 MHz.

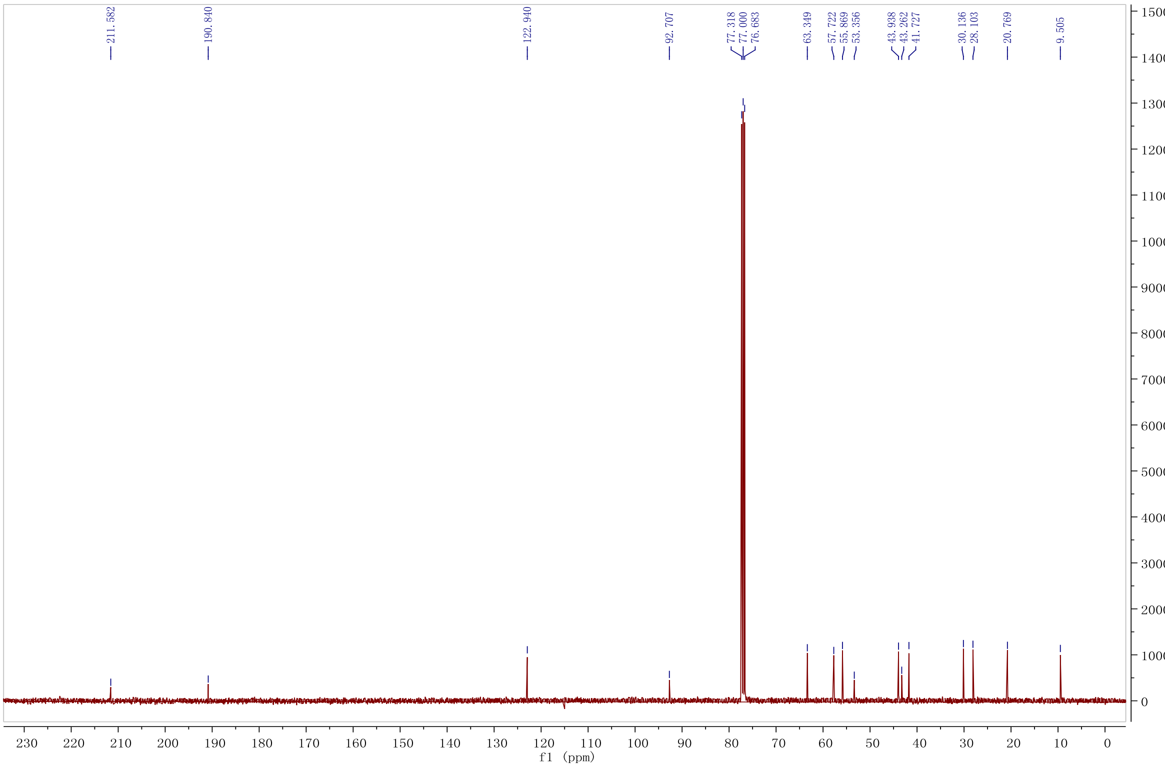

Figure S28. ^{13}C NMR spectrum of anhydroarthrosporone (**4**) in $CDCl_3$, 100 MHz.

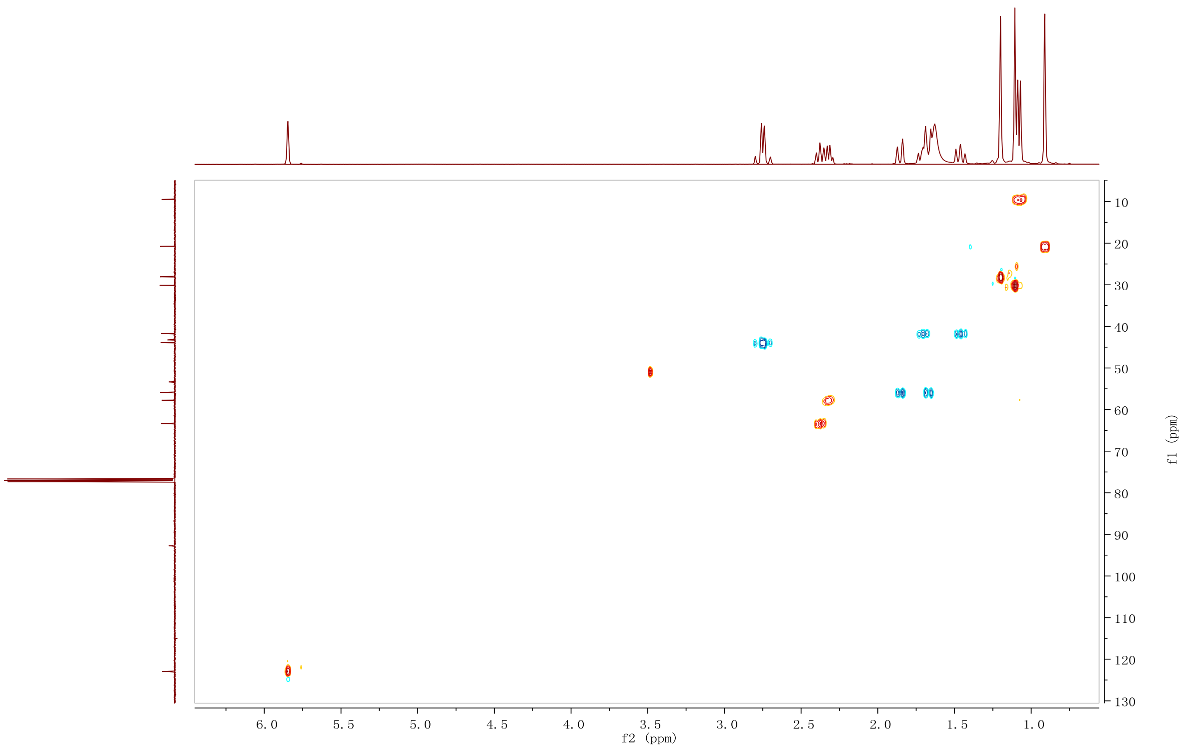

Figure S29. HMQC spectrum of anhydroarthrosporone (**4**).

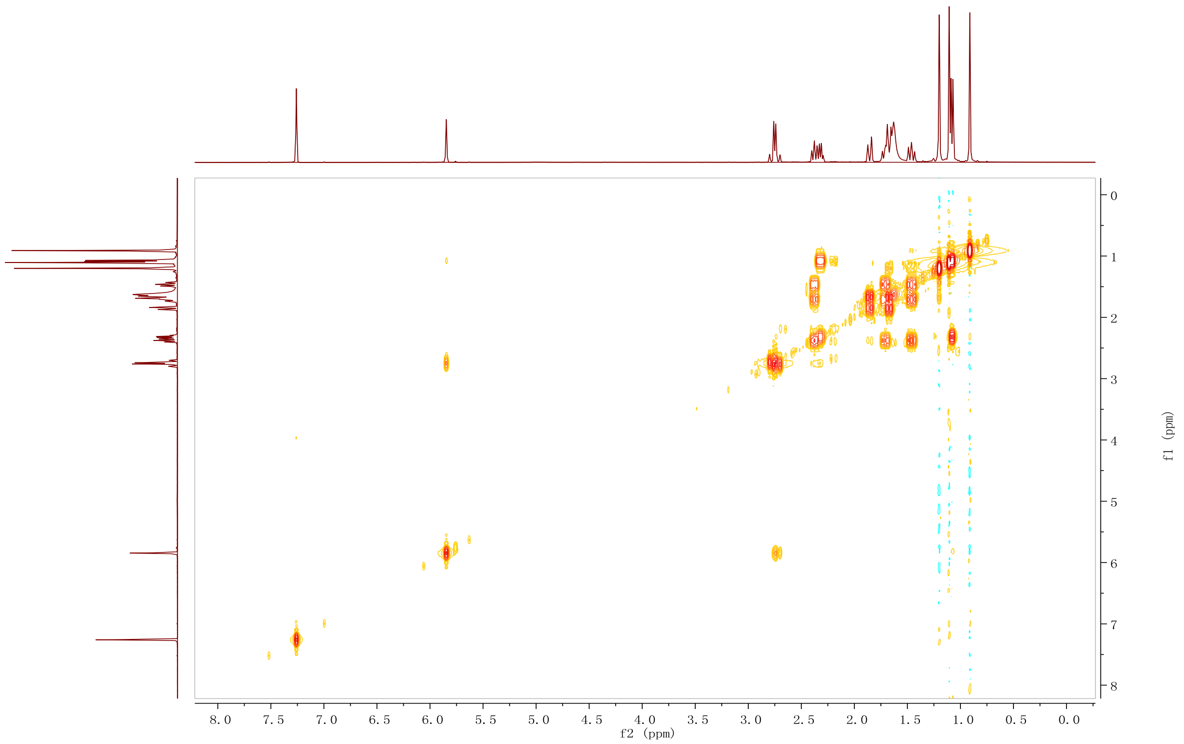

Figure S30. ^{1}H–^{1}H COSY spectrum of anhydroarthrosporone (**4**).

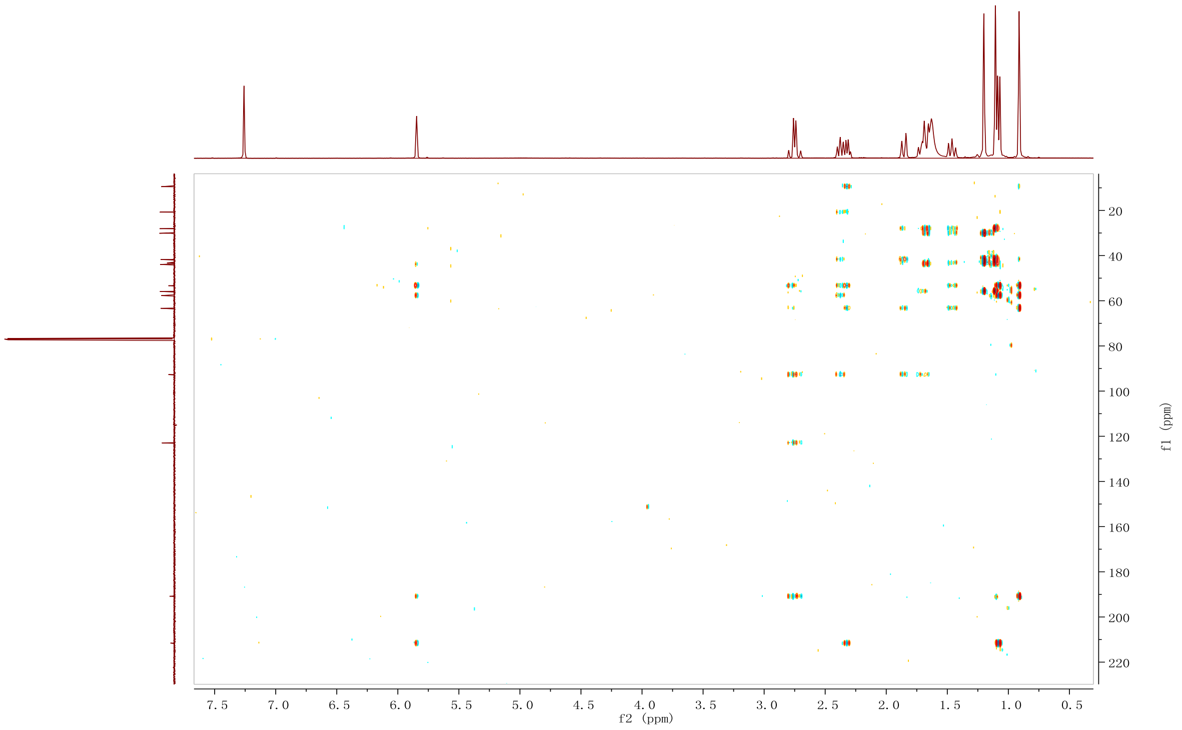

Figure S31. HMBC spectrum of anhydroarthrosporone (**4**).

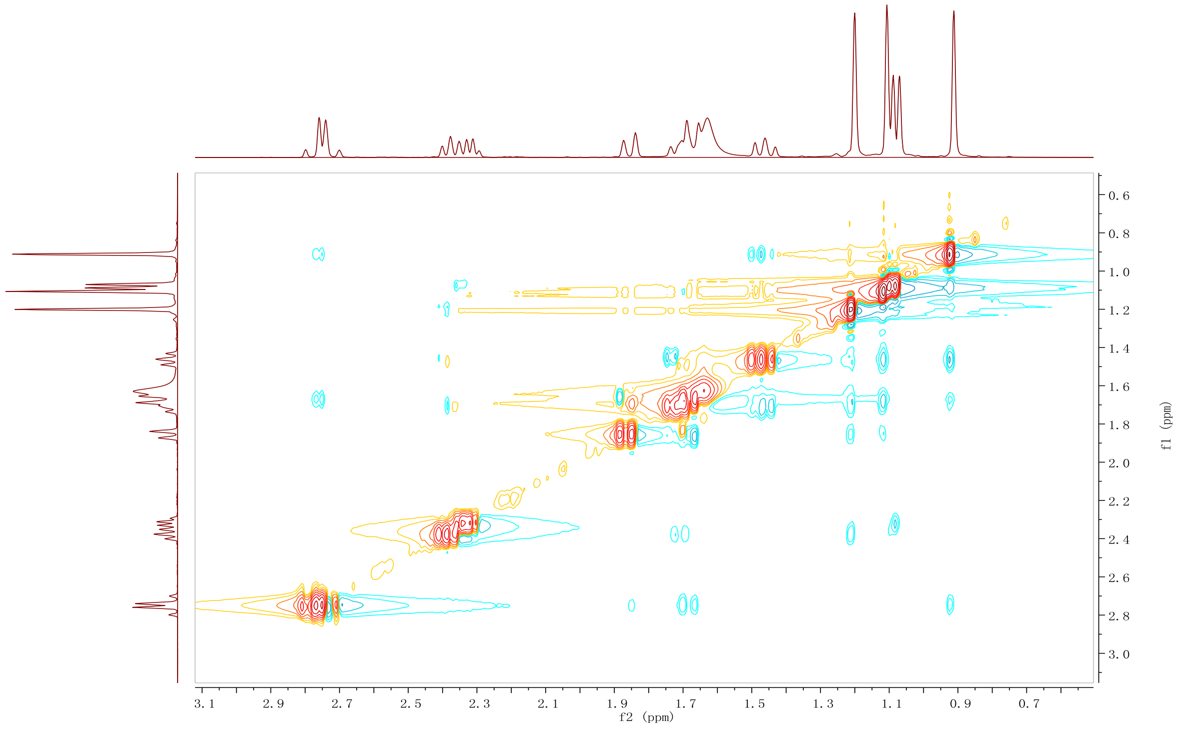

Figure S32. NOESY spectrum of anhydroarthrosporone (**4**).

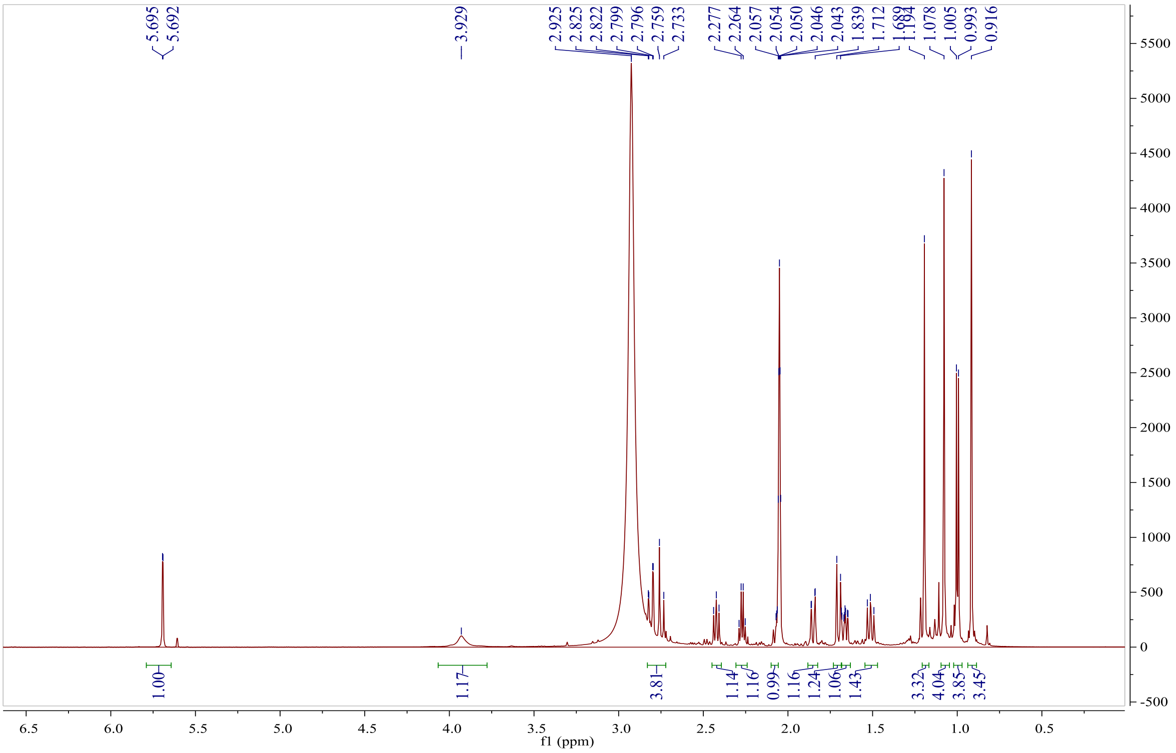

Figure S33. ^{1}H NMR spectrum of anhydroarthrosporone (**4**) in Acetone-*d*6, 600 MHz.

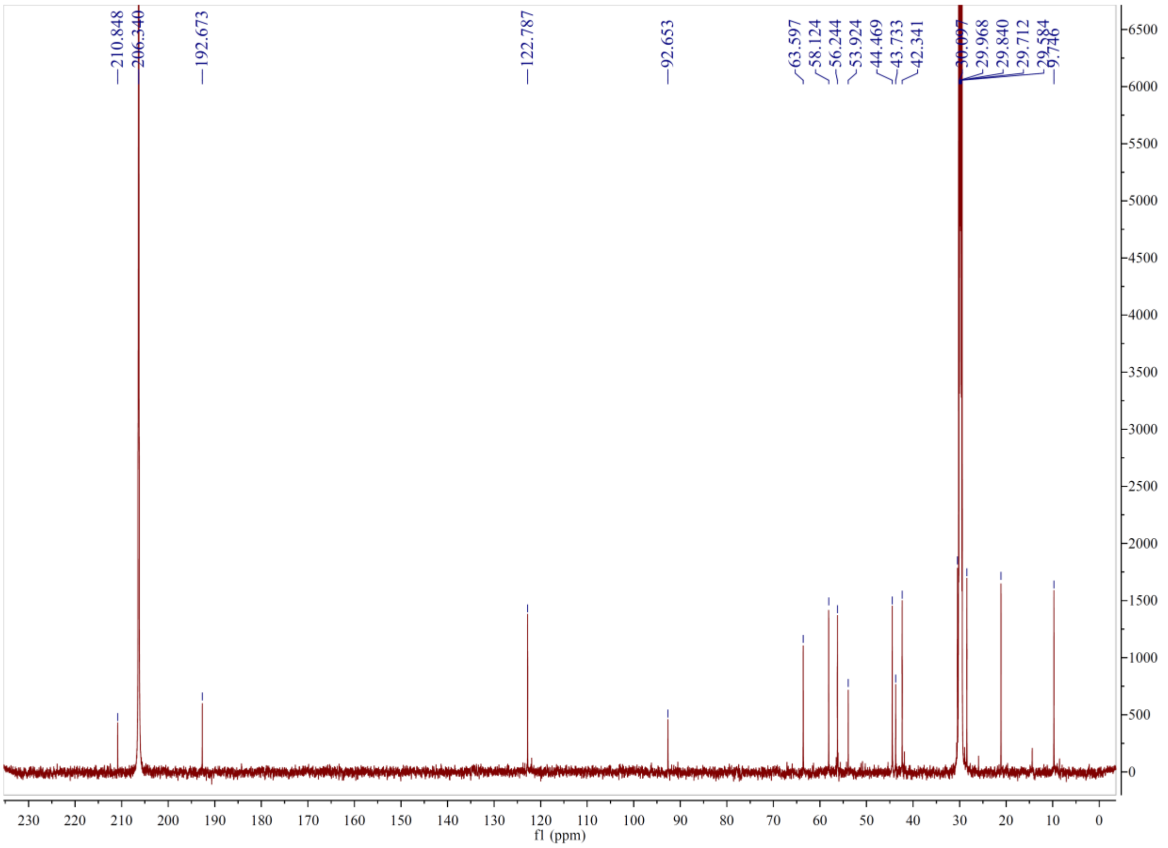

Figure S34. ^{13}C NMR spectrum of anhydroarthrosporone (**4**) in Acetone-*d*6, 150 MHz.
